# Epigenetic alteration contributes to the transcriptional reprogramming in T-cell prolymphocytic leukemia

**DOI:** 10.1038/s41598-021-87890-9

**Published:** 2021-04-15

**Authors:** Shulan Tian, Henan Zhang, Pan Zhang, Michael Kalmbach, Jeong-Heon Lee, Tamas Ordog, Paul J. Hampel, Timothy G. Call, Thomas E. Witzig, Neil E. Kay, Eric W. Klee, Susan L. Slager, Huihuang Yan, Wei Ding

**Affiliations:** 1 Division of Biomedical Statistics and Informatics, Department of Health Sciences Research, Mayo Clinic, Rochester, MN USA; 2 Department of Urology, Mayo Clinic, Rochester, MN USA; 3 Division of Information Management and Analytics, Department of Information Technology, Mayo Clinic, Rochester, MN USA; 4 Division of Experimental Pathology and Laboratory Medicine, Department of Laboratory Medicine and Pathology, Mayo Clinic, Rochester, MN USA; 5 Department of Physiology and Biomedical Engineering, Mayo Clinic, Rochester, MN USA; 6 Division of Hematology, Mayo Clinic, Rochester, MN USA; 7 Present Address: Illinois Informatics Institute, University of Illinois at Urbana-Champaign, Urbana, IL USA

**Keywords:** Computational biology and bioinformatics, Genetics

## Abstract

T cell prolymphocytic leukemia (T-PLL) is a rare disease with aggressive clinical course. Cytogenetic analysis, whole-exome and whole-genome sequencing have identified primary structural alterations in T-PLL, including inversion, translocation and copy number variation. Recurrent somatic mutations were also identified in genes encoding chromatin regulators and those in the JAK-STAT signaling pathway. Epigenetic alterations are the hallmark of many cancers. However, genome-wide epigenomic profiles have not been reported in T-PLL, limiting the mechanistic study of its carcinogenesis. We hypothesize epigenetic mechanisms also play a key role in T-PLL pathogenesis. To systematically test this hypothesis, we generated genome-wide maps of regulatory regions using H3K4me3 and H3K27ac ChIP-seq, as well as RNA-seq data in both T-PLL patients and healthy individuals. We found that genes down-regulated in T-PLL are mainly associated with defense response, immune system or adaptive immune response, while up-regulated genes are enriched in developmental process, as well as WNT signaling pathway with crucial roles in cell fate decision. In particular, our analysis revealed a global alteration of regulatory landscape in T-PLL, with differential peaks highly enriched for binding motifs of immune related transcription factors, supporting the epigenetic regulation of oncogenes and genes involved in DNA damage response and T-cell activation. Together, our work reveals a causal role of epigenetic dysregulation in T-PLL.

## Introduction

T cell prolymphocytic leukemia (T-PLL) is a rare disease with a male predominance, representing ~ 2% of the mature lymphocytic leukemias in adults^1,2^. T-PLL is currently considered incurable. As an aggressive disease, T-PLL patients show a rapid clinical course with a median survival of < 1 year^3^. An overall response of > 90% can be observed in immunotherapy with the monoclonal anti-CD52 antibody alemtuzumab, but relapse is inevitable due to resistance^1,2^. Relapsed T-PLL typically progresses rapidly with final demise. Thus, the prognosis for patients diagnosed with T-PLL remains far from satisfactory^4^. Recently, combination therapy with epigenetic agents such as pan-histone deacetylase inhibitors (HDACi) vorinostat/romidepsin was shown to overcome alemtuzumab resistance to some extent^1^, suggesting the involvement of epigenetic mechanism.

Cytogenetic analysis^2,3^, array comparative genomic hybridization^2,4^, whole-exome sequencing (WES) and whole-genome sequencing (WGS)^4,5^ have revealed primary structural alterations in T-PLL, including inv(14)(q11q32) and t(14;14)(q11;q32) that juxtapose the oncogene *TCL1A* at 14q32 to the T cell receptor alpha and delta (TRA/TRD) locus at 14q11, and less frequently, t(X;14)(q28;q11) that juxtaposes the oncogene *MTCP1* (Xq28) to the TRA/TRD locus too. In addition, structural variations are commonly seen on chromosome 8, such as trisomy of 8q^2–4^, copy number gains on 8p^3^, i(8)(q10)^2–4^ and der(8)t(8;8)^3^. Copy number variations (CNVs) were also observed on several other chromosomes^2–4^.

WGS^4,5^, WES^4,5^ and sequencing of PCR amplicons^2^ have uncovered recurrent somatic events, including deletions and missense mutations in the tumor suppressor gene *TP53*^2^, and deletion, missense and frameshift mutations in another tumor suppressor, *ATM*, which is a key regulator of DNA damage response^2,4^. ATM is a serine/threonine kinase responsible for the phosphorylation of IκBα, which functions as an inhibitor of the NF-κB transcription factor (TF)^6^. A recent genomic and transcriptome analysis has revealed a central role of *ATM* aberrations in T-PLL pathogenesis^5^, likely through its role in DNA damage repair^4,5^. In addition, largely mutually exclusive gain-of-function mutations were identified in *IL2RG*, *JAK1*, *JAK3* and *STAT5B* in the JAK-STAT signaling pathway, which were found to enhance cytokine-independent cell proliferation^4^.

Finally, mutations in several epigenetic genes encoding chromatin regulators were also reported, including missense mutations in the histone methyltransferase *EZH2*, which deposits H3K27 methylation^4^, in *TET2*, which hydroxylates 5-methylcytosine to 5-hydroxymethylcytosine^3^, and in *CREBBP*, which encodes the histone acetyltransferase CREB-binding protein^4^. Despite the identification of recurrent somatic variants, CNVs and genomic rearrangements, the complex mechanism of T-PLL pathogenesis is only partially understood. So far, histone modifications were analyzed only in promoter regions of two genes for repressive marks H3K9me3 and H3K27me3, and for pan-H4 acetylation using ChIP-qPCR^1^. Thus, a lack of genome-wide epigenetic data has limited the investigation of the involvement of epigenetic mechanisms in this deadly disease.

In this study, using ChIP-seq data for H3K4me3 and H3K27ac that define promoters and active enhancers, respectively, we found global alterations of regulatory regions in T-PLL, with over 10% of the peaks showing differential occupancy between T-PLL and normal. Through total RNA and mRNA sequencing we identified 1672 and 2364 genes differentially expressed between T-PLL and normal samples, including *TCL1A*, *ATM* and several T-cell receptor regulators that are known to play key roles in T-PLL pathogenesis. Notably, for both histone marks, differential peaks are strongly linked to differential expression of the nearest genes, suggesting a role for epigenetic mechanism in transcriptional reprogramming in T-PLL. We characterized several well-known genes dysregulated in T-PLL in detail and demonstrated that the deregulation of those genes is associated with changes of local chromatin state at their promoters, enhancers, or both. These results have elucidated major epigenetic events and their possible causal roles in T-PLL pathogenesis.

## Methods

### T-PLL patients and healthy subjects

The Mayo Clinic patients who had a clinical diagnosis of T-PLL and donated their blood samples to Mayo Clinic chronic lymphocytic leukemia (Mayo Clinic IRB 1827–00) and lymphoma (Mayo Clinic IRB 118) tissue banks were used in this study. After screening all T-PLL samples in the above tissue banks, only six were stored with more than 50 million fresh peripheral blood mononuclear cells (PBMCs) that were used for ChIP-seq in this study (Table 1). Four of the six samples were used for total RNA sequencing; two of the six samples plus another three samples were used for mRNA sequencing. All but one of the nine T-PLL cases had the characteristic rearrangement involving *TCL1A*. The Mayo Clinic blood bank provided peripheral blood samples from three age-matched healthy individuals for ChIP-seq and total RNA sequencing and another four for mRNA sequencing (Mayo Clinic IRB 07-05543). Samples were collected with written consent and approval from the institutional review board at Mayo Clinic. We used PBMCs from the T-PLL samples that are over 90% leukemic T cells. For the normal controls, we selected CD3 + enriched PBMCs. All experiments were performed in accordance with relevant guidelines and regulations.

**Table 1:** Clinical information about the nine T-PLL cases.

ID	Date of sample	Treatment*	Time^+^	Phenotype	Key FISH results
P1	1/28/16	CampathX2, romidepsin	< 1 year	CD3+ , CD4 + , CD45RA+ , CD45RO-, CCR7+ , CD69+	88.5% of nuclei had a rearrangement involving *TCL1A*
P2	9/29/13	Untreated, observed from 2009 to 2013	4 years	CD3 , CD8+ , CD45RO-, CD45RA+ , CCR7 +	35.5% nuclei with inv(14) or t(14;14) involving *TCL1A*, also with 11q- in 60% nuclei
P3	4/13/16	Campath, methotrexate, pentostatin, pralatrexate. CHOP, Benda	< 2 months	CD3+ , CD4+ , CD45RA+ , CCR7+ , CD62L+	74% nuclei has *TCL1A* rearrangement
P4	2012	Untreated, found in 2008, observed to 2012	4 years	CD3 + , CD4 + , CD45RA+ , CCR7 + , CD62L+	70% nuclei had rearrangement involving *TCL1A*, either inv(14) or t(14;14)
P5	7/14/14	Untreated, but in half a year required therapy for skin rash and cytopenia	0.5 year	CD3+ , CD8+ , CD45RO+ , CCR7+ , CD62L+	14q32.1 (*TCL1A* sep), abnormal in 90.0% nuclei
P6	9/11/15	Untreated, required therapy in 2 months	2 months	CD3+ , CD4 + , CD45RO+ , CCR7+ , CD62L +	82% of nuclei had a rearrangement involving *TCL1A*, 93.0% of nuclei with additional copies of 5′TCRB region, and 68.0% of nuclei with an additional copy of *MYC* (8q24)
P7	1/23/12	Untreated, with 2 months started alemtuzumab and pentostatin, followed by Hyper-CVAD, Fludarabine and cyclophosphamide	2 months	CD3+ , CD8 + , CD45RA+ , CCR7+ , pCD62L + , CD95+	98% nuclei rearrangement involved *TCL1A*, 83% nuclei had three signals for *MYC* (at 8q24.1), 17p- and del (11q)
P8	7/10/10	Untreated, within 5 months, started alemtuzumab and pentostatin	5 months	CD3+ , CD4 + , CD45RO+ , CCR7+ , pCD62L + , pCD95+	T*CL1* abnormal (83.5%)
P9	11/1/04	Untreated, within 2 months, started alemtuzumab, pentostatin and cyclophosphamide	2 months	CD3 + , CD4+ , CD45RA+ , CCR7 + , pCD62L+ , pCD95+	46,X,-X,t(6;6)(q27;q15),inv(14)(q13q24), + mar[15]/

*Treatment history prior to sample collection. Patient P1 received a three-month romidepsin treatment, which was discontinued one month prior to sample collection.

^+^Time from diagnosis to therapy.

### RNA preparation and sequencing

For three normal (N1-N3) and four (P1, P3, P5 and P6) of the nine T-PLL patients, total RNA was extracted with Qiagen miRNeasy Mini Kit on the QIAcube according to manufacturer instructions. Total RNA libraries were prepared using Illumina TruSeq Stranded Total RNA Sample Prep Kit. For the other four normal (N4-N7) and five T-PLL patients (P2, P4, P7-P9), mRNA libraries were prepared using Illumina TruSeq RNA Sample Prep Kit v2. Libraries were sequenced from both ends to 101 bases on a HiSeq2000 platform through the Mayo Clinic Medical Genome Facility.

### Chromatin immunoprecipitation and sequencing

H3K4me3 is largely located in the promoters and H3K27ac in the active enhancers. These two histone marks are widely used to map gene regulatory regions. We thus performed ChIP using anti-H3K27ac antibody (Abcam, Cat. # ab4729) and in-house generated anti-H3K4me3 antibody, as previously described^7^. We validated the H3K4me3 antibody by dot blotting with peptide, ChIP-qPCR with known positive and negative targets, and ChIP-seq^7^. ChIP-seq libraries were prepared from about 5 ng ChIP and input DNA using the ThruPLEX DNA-seq Kit V2 (Rubicon Genomics, Ann Arbor, MI). ChIP enrichment was validated using real-time PCR. Libraries were sequenced from both ends to 51 or 101 bases on a HiSeq4000 platform through the Mayo Clinic Medical Genome Facility.

### RNA-seq data analysis

Raw reads were aligned to the hg19 reference genome using the spliced transcripts alignment to a reference program (STAR, v2.5.2b, https://github.com/alexdobin/STAR)^8^ Read counts per gene were estimated for the Ensembl gene annotation, using the featureCounts tool in the Subread package (v1.5.1, https://subread.sourceforge.net)^9^. Gene expression was quantified as reads per kilobase of exon per million mapped reads (RPKM). Protein-coding genes differentially expressed between T-PLL and normal were identified using the edgeR package (v3.16.5, https://bioconductor.org/packages/edgeR)^10^ at the cutoff of 5% false discovery rate (FDR) and threefold change. Briefly, genes without reads in all libraries were excluded; for the remaining genes, raw read counts were normalized using the trimmed mean of M-values (TMM) method within edgeR. We used conditional maximum likelihood method within edgeR to estimate common negative binomial dispersion across genes, which is proved to be effective for small sample size.

To functionally categorize the differentially expressed genes, pathway and gene set enrichment analyses were done separately for the genes up- and down-regulated in T-PLL. For pathway analysis, we used the ReactomePA package (v1.18.1, https://bioconductor.org/packages/release/bioc/html/ReactomePA.html)^11^ that takes the Reactome database^12^. For gene set enrichment analysis, we used the GOstats package (v2.40.0, https://bioconductor.org/packages/release/bioc/html/GOstats.html)^13^, which calculates p value for the overrepresentation of gene sets in Gene Ontology (GO) Biological Process and Molecular Function.

To highlight the gene expression changes between T-PLL and normal and the level of heterogeneity within T-PLL, we performed unsupervised clustering of expression levels for the top 10,000 protein-coding genes, using the pheatmap package (v1.0.8, https://cran.r-project.org/web/packages/pheatmap/) in R. Gene expression RPKM was log_2_ transformed and quantile normalization using limma package (v 3.30.13, https://bioconductor.org/packages/release/bioc/html/limma.html). The 10,000 genes were selected from autosomes that had RPKM values ≥ 0.5 in at least one of the samples and showed the largest variation across samples. Total RNA and mRNA sequencing data were separated when used in the above differential analysis and unsupervised clustering.

### ChIP-seq data analysis

ChIP-seq data were analyzed with the HiChIP pipeline with minor modifications^14^. In brief, reads were mapped to the hg19 reference genome using Burrows-Wheeler Aligner (BWA, v0.7.10, https://bio-bwa.sourceforge.net)^15^. After excluding read pairs with both ends being mapped uniquely or mapped to multiple locations, the remaining reads were re-mapped with Genomic Short-read Nucleotide Alignment Program (GSNAP, gmap_2015_0723, http://research-pub.gene.com/gmap/archive.html)^16^ to improve mapping rate. BWA and GSNAP alignments were combined and uniquely mapped pairs of reads with mapping quality ≥ 20 were extracted. Duplicates were removed using Picard (v1.67, https://broadinstitute.github.io/picard/). To visualize signal in the Integrative Genomics Viewer (IGV, v2.3.93, http://software.broadinstitute.org/software/igv/)^17^, we used BEDTools^18^ and in-house scripts to generate per-million read count over 200-bp sliding windows with 20-bp step size. H3K4me3 and H3K27ac peaks were identified using model-based analysis of ChIP-seq (MACS, v2.0.10, https://pypi.org/project/MACS2/)^19^, at the cutoff of ≤ 1% FDR and ≥ twofold change over the input. Peaks overlapping the blacklisted regions were excluded from further analysis. The lists of blacklisted regions were downloaded from http://hgdownload.cse.ucsc.edu/goldenPath/hg19/encodeDCC/wgEncodeMapability/wgEncodeDacMapabilityConsensusExcludable.bed.gz and http://mitra.stanford.edu/kundaje/akundaje/release/blacklists/hg19-human/Duke_Hg19SignalRepeatArtifactRegions.bed.gz.

To perform unsupervised clustering for H3K4me3 and H3K27ac peaks, we first merged peaks if they were overlapped by at least 1 bp. For each merged peak in each sample, we estimated the number of mapped pairs in the IP and input using BEDTools. Read counts in IP were input-subtracted, normalized to 10 M mapped reads, log_2_ transformed and quantile normalized. We retained merged peaks that were from autosomal chromosomes and present in at least two samples. For H3K27ac, we focused on merged peaks that are located in the enhancers and removed those that overlapped the TSS ± 2.5 kb regions from protein-coding genes, as described^20,21^. The top 20,000 merged peaks with the largest variation across samples were retained for unsupervised clustering. H3K4me3 peaks were processed similarly, except that we focused on those in the promoters of protein-coding genes, defined as the TSS ± 2 kb regions^22^. To view the H3K4me3 and H3K27ac changes for genes up- or down-regulated in T-PLL, we generated aggregate per-million signal density profiles over TSS ± 2 kb using ngs.plot tool (v2.63, https://github.com/shenlab-sinai/ngsplot)^23^.

We used the DiffBind R package (v2.14.0, https://bioconductor.org/packages/release/bioc/html/DiffBind.html)^24^ to identify the changes of H3K27ac and H3K4me3 between the six T-PLL and three normal subjects, and between the four T-PLL samples and three normal controls that also have total RNA sequencing data. Only merged peaks that were present in ≥ 2 samples were included. Data were TMM normalized and differential peaks were identified using the edgeR method within DiffBind, at the cutoff of ≤ 5% FDR and ≥ twofold change. Differential peaks were assigned to the nearest genes using the closestBed command in BEDTools.

### Copy number alteration (CNA) analysis

CNA analysis was done according to^25^ with minor modifications. Instead of using 1-Mb windows with 500-kb overlap and an absolute z-score cutoff of >  = 2, we split each autosome into 100-kb windows with a step size of 10 kb, and accordingly used a more stringent cutoff of absolute z-score >  = 3. For each of the normal and T-PLL cases, alignments from H3K4me3, H3K27ac and input libraries were combined and those in the peaks and blacklisted regions (see above) were excluded. BEDTools was used to estimate per window read pairs in each sample. Windows with no (6.37% of the windows) or less than 100 read pairs (0.87% of the windows) were excluded. The coverage for the remaining windows was calculated as: Coverage = (Window read counts scaled to 10 M)/(Effective window size in kb), where effective window size does not include the base pairs overlapping peaks and blacklisted regions. The coverage in each of the six T-PLL was normalized to the average of the median coverage from the three normal, and log2 transformed. Z score was calculated using the above median-normalized coverage in each PLL sample. A copy number gain or loss was inferred if the z score >  = 3 or <  = − 3, respectively. The differential H3K4me3 and H3K27ac peaks were assigned the CNA status of the nearest 100-kb window with the shortest distance between peak center and window middle point.

### TF motif enrichment analysis

For differential H3K4me3 and H3K27ac peaks, we identified the nearest open chromatin regions by intersecting with ATAC-seq peaks from blood^26,27^ (see below). Sequences from ATAC-seq peak center ± 50 bp were extracted and enrichment of known TF motifs was identified using the Homer package (v4.10, http://homer.ucsd.edu/homer/).

### Public epigenomics data

We downloaded H3K27ac ChIP-seq reads alignments and MACS peaks from the Roadmap reference epigenomes^28^, which are available at http://egg2.wustl.edu/roadmap/data/byFileType. Signal tracks were then generated from alignments using the HiChIP pipeline^14^. Raw sequence data for H3K27ac ChIP-seq in four normal B-cell samples were downloaded from SRA, under the accession GSM998996 and GSM998997^29^, GSM2386722^30^, and GSM1027287^31,32^. H3K27ac signal tracks (tdf or bw files) were downloaded from GEO for seven mantle cell lymphoma (GSM1703947, GSM1703949, GSM1703951, GSM1703953, GSM2760324, GSM2760326, and GSM2760328)^33,34^, three small lymphocytic lymphoma (GSM1703955, GSM1703957, and GSM1703959)^33^, and seven high-grade B-cell lymphoma patient samples (GSM1703933, GSM1703935, GSM1703937, GSM1703939, GSM1703941, GSM1703943, and GSM1703945)^33^, as well as 10 adult T-cell leukemia/lymphoma (ATL) patients (GSE85695)^35^. ATAC-seq data were downloaded for 14 human primary blood cell types (GSE74912, 50 samples)^26^ and 32 immune cell populations (GSE118189, 97 samples)^27^. Peaks were identified through the HiChIP pipeline, and those with FDR <  = 1e−05 were retained. Catalog of super-enhancers from 86 cell and tissue types was obtained from^36^. Finally, to identify enhancer for *MYC*, we used the promoter capture Hi-C (CHi-C) data from 17 blood cell types^37^; regions of significant interactions with CHiCAGO score ≥ 5 are available at https://osf.io/u8tzp/.

## Results

### Transcriptional dysregulation in T-PLL

**Figure 1:**
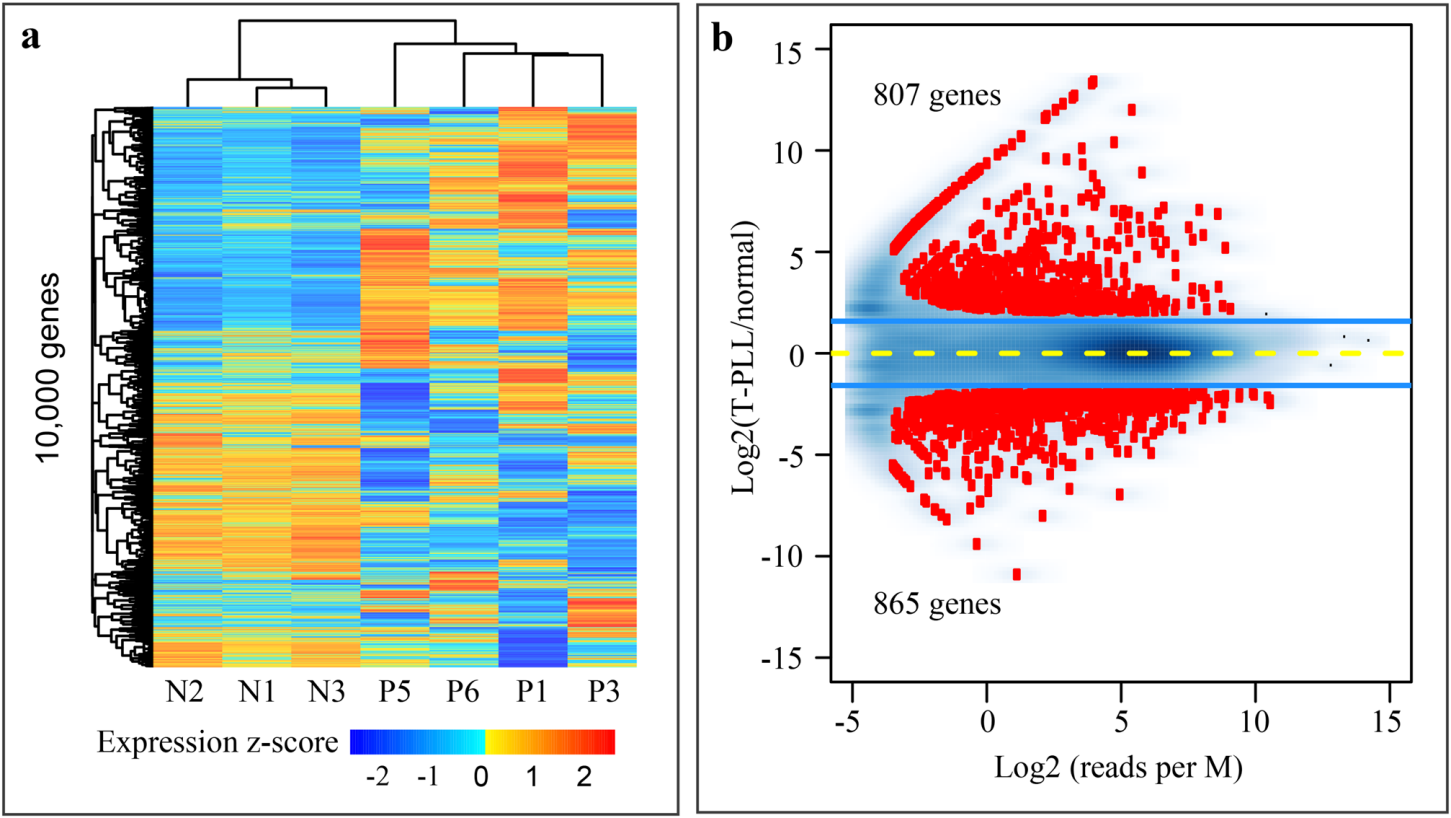
Gene expression changes in T-PLL. (**a**) Unsupervised clustering of expression level from the top 10,000 genes. Gene expression was quantified by total RNA sequencing as RPKM values and transformed to log_2_ scale with an offset of 0.1, following by quantile normalization. These genes were selected from autosomes that had RPKM ≥ 0.5 in at least one of the samples and showed the largest variation across samples. Unlike the three normal, the T-PLL cases showed remarkable heterogeneity in expression profile. N1-N3, normal individuals; P1, P3, P5 and P6, T-PLL patients. (**b**) Protein-coding genes differentially expressed between T-PLL and normal. Raw reads were aligned to the hg19 reference genome using STAR and the number of reads in exons per gene was estimated with featureCounts. The 1672 (807 + 865) differentially expressed genes were identified using edgeR at the cutoff of 5% FDR and threefold change. Y-axis, fold-change at the log_2_ scale; x-axis, sum of the normalized read count per M from the normal and T-PLL group.

We performed total RNA sequencing in PBMCs from four T-PLL patients (P1, P3, P5, and P6) and three healthy individuals (N1-N3), and mRNA sequencing for another five T-PLL (P2, P4, and P7-P9) and four normal (N4-N7). Of the nine T-PLL patients, P1 and P3 had already received treatment when samples were collected, while the other seven patients were treatment naive (Table 1). In total RNA sequencing, a total of 12,375 protein-coding genes had RPKM ≥ 0.5 in at least one of the samples. The top 10,000 most variable genes were used in unsupervised clustering, which revealed distinctive differences between T-PLL and normal samples (Fig. 1a). We observed noticeable heterogeneity of gene expression within T-PLL. In mRNA sequencing, a total of 13,728 protein-coding genes had RPKM ≥ 0.5 in at least one of the samples. Unsupervised clustering using the top 10,000 most variable genes revealed a similar pattern of expression changes between T-PLL and normal and heterogeneity across T-PLL patients (Supplemental Fig. 1a).

Using total RNA sequencing, we identified 807 and 865 genes that were up- and down-regulated, respectively, in T-PLL compared to normal (Fig. 1b). In addition, mRNA sequencing revealed 1466 up- and 898 down-regulated genes in T-PLL (Supplemental Fig. 1b). Of those, 280 up- and 279 down-regulated genes were shared between the two RNA-seq datasets. mRNA sequencing identified 1.8 × as many up-regulated protein-coding genes in T-PLL compared to total RNA sequencing. This discrepancy is likely due to difference in sequencing depth, platform-specific bias of the two RNA-seq protocols, and the heterogeneity among T-PLL cases. To gain a better understanding of the possible causes, we first estimated the sequencing depth by counting the number of reads mapped to all 57,773 genes and to the subset of 20,327 protein-coding genes. While normal samples had largely comparable number of reads mapped to protein-coding genes in both datasets, T-PLL samples indeed had lower coverage of protein-coding genes in total RNA sequencing (17.87–37.13 M) than in mRNA sequencing (41.52–52.74 M) (Supplemental Fig. 2a). Next, we assessed expression level for the protein-coding genes up-regulated in T-PLL between the two datasets, using gene maximum RPKM values across both normal and T-PLL cases. Up-regulated genes identified in both datasets showed similar levels of expression (Mann–Whitney U test *p* = 0.09, left panel in Supplemental Fig. 2b). Not surprisingly, for the genes identified as up-regulated in T-PLL by mRNA sequencing alone, they had much higher expression levels in mRNA sequencing than in total RNA sequencing (*p* < 2.2e−16, middle panel in Supplemental Fig. 2b), and vice versa (*p* < 2.2e−16, right panel in Supplemental Fig. 2b). It is known that genes with low counts are less likely to be significant in differential expression test due to a lack of statistical power^38,39^.

For both datasets, Gene Ontology analysis indicated a remarkable enrichment of the down-regulated genes in immune response (Table 2 and Supplemental Table 1). Similarly, pathway enrichment analysis using the Reactome database revealed enrichment in immune and inflammatory responses, including the “chemokine receptors”, “immune system”, and “immunoregulatory interactions between a lymphoid and a non-lymphoid cell” (Table 2 and Supplemental Table 1). On the other hand, Gene Ontology analysis revealed that the up-regulated genes were enriched in “WNT-protein binding” and “WNT-activated receptor activity”, as well as in many other categories of developmental related biological processes (Supplemental Tables 2 and 3). This result indicates a similar role for WNT signaling network in T-PLL, as documented in B-cell chronic lymphocytic leukemia^40–43^ and T-lineage acute lymphoblastic leukemia^44^.

**Table 2:** Enriched pathways and GO terms for genes down-regulated in T-PLL.

Name	No. gene	Q value	Source
Immune System	214	7.34E−26	Reactome
Immunoregulatory interactions between a Lymphoid and a non-Lymphoid cell	43	1.31E−21	Reactome
Neutrophil degranulation	70	8.87E−14	Reactome
Innate Immune System	129	1.96E−13	Reactome
Adaptive Immune System	87	2.47E−09	Reactome
Cytokine Signaling in Immune system	80	7.29E−09	Reactome
Costimulation by the CD28 family	17	3.58E−06	Reactome
PD-1 signaling	10	4.66E−06	Reactome
Chemokine receptors bind chemokines	13	2.19E−05	Reactome
Interferon gamma signaling	18	2.69E−05	Reactome
Immune response (GO:0006955)	185	4.48E−38	GO
Immune system process (GO:0002376)	242	4.48E−38	GO
Cell activation (GO:0001775)	115	2.29E−24	GO
Defense response (GO:0006952)	162	5.41E−27	GO
Regulation of immune system process (GO:0002682)	158	3.03E−31	GO
Leukocyte activation (GO:0045321)	97	1.89E−21	GO
Regulation of immune response (GO:0050776)	110	4.00E−23	GO
Immune effector process (GO:0002252)	76	8.04E−13	GO
Leukocyte mediated immunity (GO:0002443)	41	4.75E−10	GO
Myeloid leukocyte activation (GO:0002274)	30	2.84E−10	GO

Gene expression was quantified by total RNA sequencing for three normal (N1-N3) and four T-PLL (P1, P3, P5, and P6).

**Figure 2:**
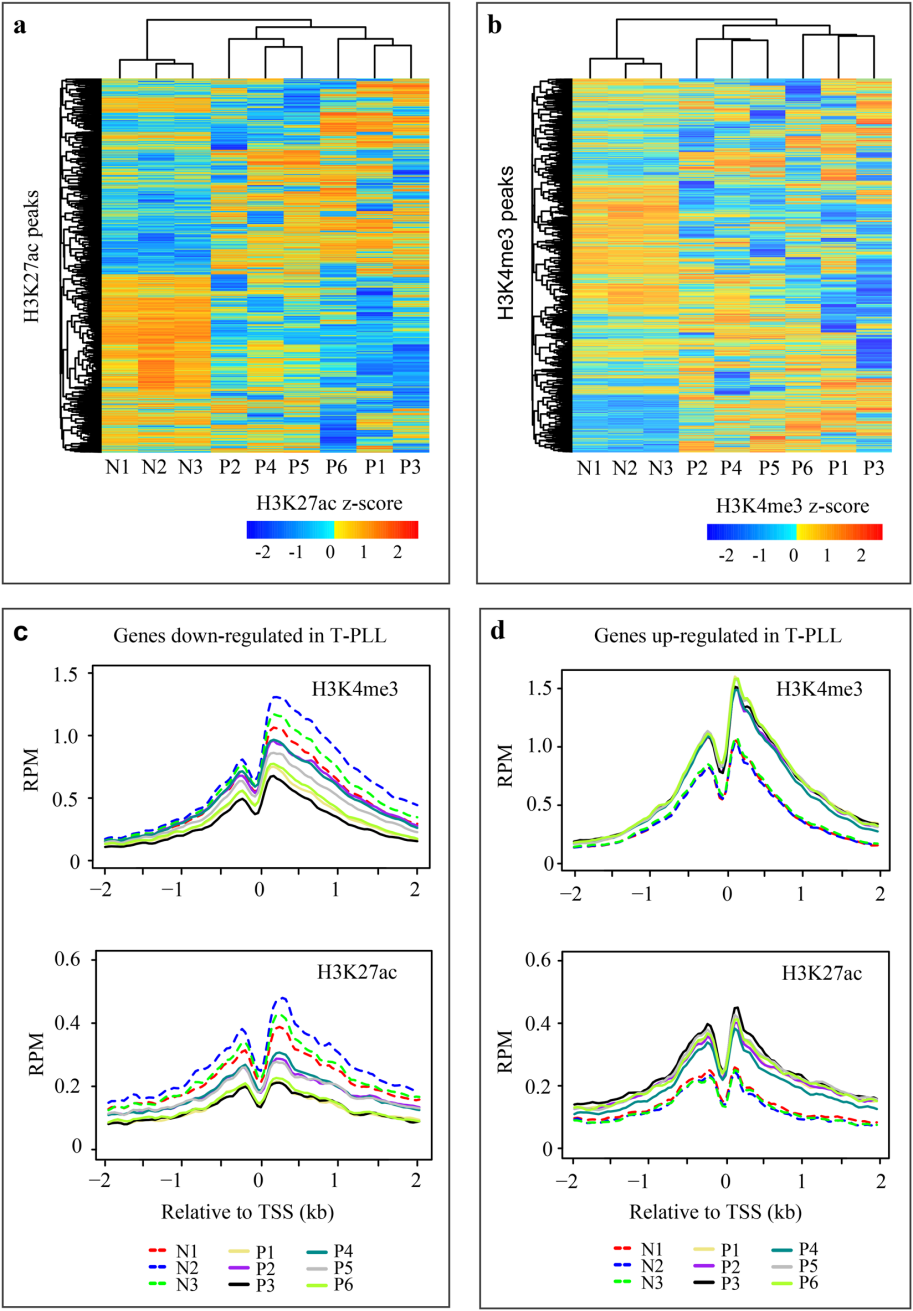
Global alteration of regulatory regions in T-PLL. (**a**) Unsupervised clustering of 20,000 H3K27ac peaks. Peaks from all nine samples were first merged if they were overlapped by at least 1 bp. For each merged peak, the input-subtracted read counts were normalized to 10 M mapped reads, log_2_ transformed and quantile normalized. The top 20,000 merged peaks were selected from autosomes that were each present in at least two samples, not in the TSS ± 2.5 kb regions, and had the largest between-sample variation. (**b**) Unsupervised clustering of 10,000 H3K4me3 peaks. The H3K4me3 peaks were processed similarly as above, except that only those in the TSS ± 2 kb regions of protein-coding genes were selected. (**c**) Average read density profile over TSS ± 2 kb for genes down-regulated in T-PLL. H3K4me3 and H3K27ac signals in 40-bp bins were estimated using the ngs.plot software. RPM, reads per M. (**d**) Average read density profile over TSS ± 2 kb for genes up-regulated in T-PLL.

**Table 3:** Association of differential H3K27ac and H3K4me3 occupancy with differential gene expression.

Mark	Region	Differential peaks			Non-differential peaks		Enrichment
		Type	Total	Linked to up-regulated gene	Total	Linked to up-regulated gene	
H3K27ac	> TSS ± 2.5 kb	Up in normal	5462	1001 (265)	35,788	1372 (323)	4.78
H3K27ac	> TSS ± 2.5 kb	Up in T-PLL	2833	583 (167)	35,788	1112 (287)	6.62
H3K27ac	≤ TSS ± 2.5 kb	Up in normal	524	212 (136)	16,866	355 (213)	19.22
H3K27ac	≤ TSS ± 2.5 kb	Up in T-PLL	543	166 (123)	16,866	270 (187)	19.1
H3K4me3	> TSS ± 2.0 kb	Up in normal	1959	272 (175)	17,596	635 (321)	3.83
H3K4me3	> TSS ± 2.0 kb	Up in T-PLL	1086	165 (102)	17,596	626 (300)	4.27
H3K4me3	≤ TSS ± 2.0 kb	Up in normal	742	212 (186)	15,775	471 (403)	9.57
H3K4me3	≤ TSS ± 2.0 kb	Up in T-PLL	148	85 (84)	15,775	509 (394)	17.8

Total RNA-seq differential expression analysis and ChIP-seq DiffBind were performed on four T-PLL (P1, P3, P5 and P6) and three normal (N1-N3). Peaks were split into those that showed differential occupancy between T-PLL and normal and others that showed no differential occupancy based on DiffBind analysis (FDR ≤ 0.05, fold-change ≥ 2). Peaks were assigned to the nearest genes. For differential peaks showing increased occupancy in normal (“Up in normal”), the number in parentheses indicates the number of up-regulated genes in normal. For differential peaks showing increased occupancy in T-PLL (“Up in T-PLL”), the number in parentheses indicates the number of up-regulated genes in T-PLL. Enrichment level is estimated as: (Number of differential peaks linked to differentially-expressed genes/total differential peaks) / (Number of non-differential peaks linked to differentially-expressed genes/total non-differential peaks).

### Global alteration of regulatory regions in T-PLL

To understand the regulatory landscape and the possible roles of epigenetic mechanisms in T-PLL pathogenesis, we generated ChIP-seq data for H3K4me3 and H3K27ac, which are mainly located in the promoters and active enhancers, respectively. To reveal the extent of global similarities in H3K4me3 and H3K27ac distribution across normal and T-PLL, we estimated pairwise Pearson correlation using normalized read counts from merged peaks present in two or more samples (Supplemental Fig. 3a,b). As expected, the three normal samples were highly similar in the occupancy of both H3K4me3 (*R* = 0.96–0.97) and H3K27ac (*R* = 0.90–0.92). On the other hand, the six T-PLL were more divergent (*R* = 0.82–0.92 for H3K4me3 and *R* = 0.65–0.86 for H3K27ac), in particular for H3K27ac, suggesting an obvious heterogeneity of epigenomic landscape. Of the six T-PLL, P1 received romidepsin treatment one month before sample collection. Romidepsin is a histone deacetylase (HDAC) class I-selective agent for HDAC1, HDAC2, HDAC3, and HDAC8. Romidepsin treatment is through to increase histone acetylation, thus altering gene expression, in particular activating tumor suppressor genes^25,45^. P1 is a non-responder to romidepsin, and was thus included in the analysis together with the other T-PLL. To support this, we assessed global enrichment of H3K27ac over the respective input (Supplemental Fig. 4a), as well as the signal levels within consensus peaks shared either by all six T-PLL or by at least two (Supplemental Fig. 4b). P1 did not show clearly excess enrichment relative to the other T-PLL. Also, we examined the expression of tumor suppressor genes^46^, revealing that P1 had the lowest median expression among the four T-PLL with total RNA sequencing data (Supplemental Fig. 4c).

**Figure 3:**
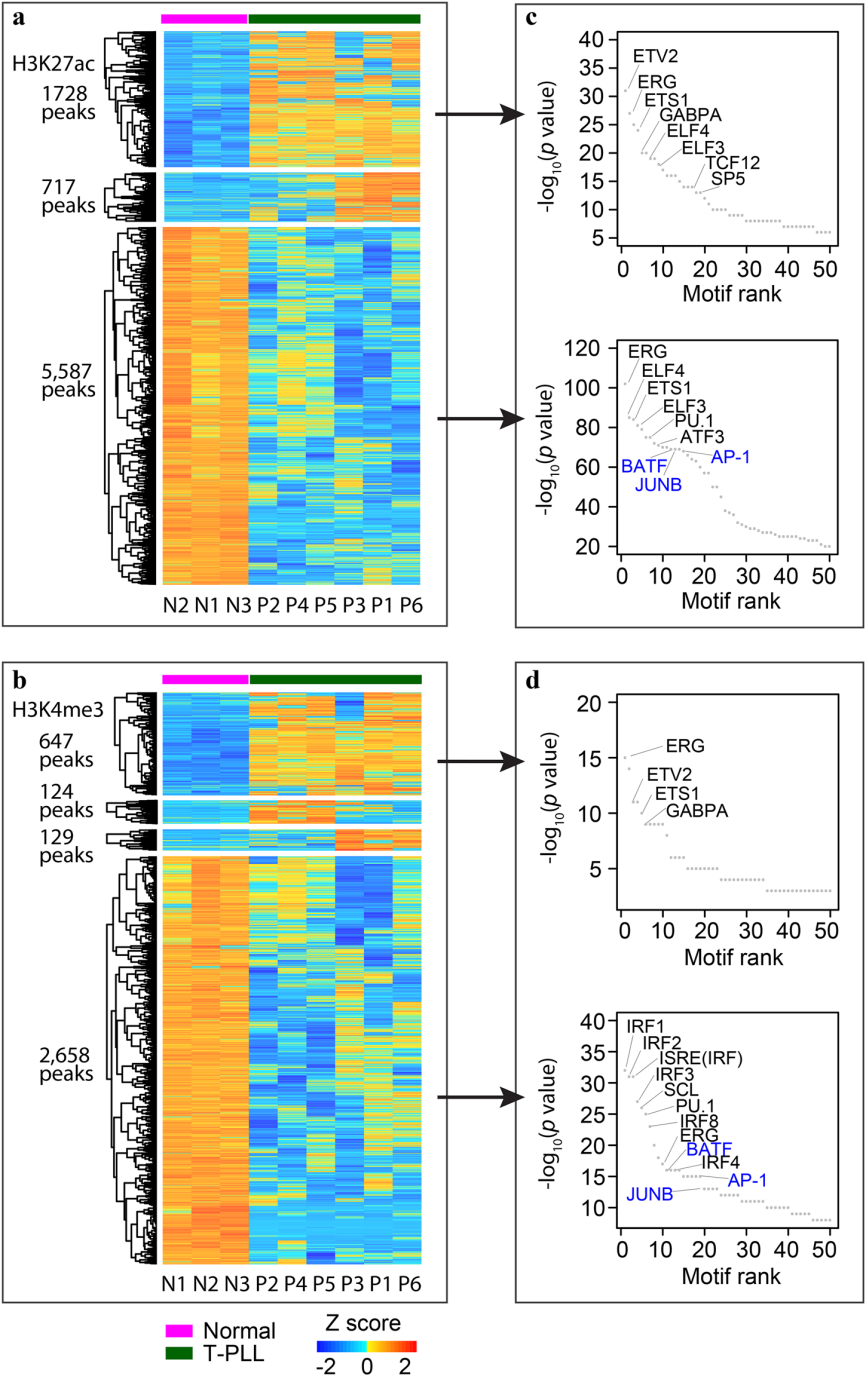
Heatmap of differential peaks and enriched TF binding motifs. (**a**) H3K27ac peaks with increased (upper panel) or decreased (lower panel) signal in T-PLL. There are 717 peaks (middle panel) that show increased occupancy in three of the T-PLL (P3/P1/P6). (**b**) H3K4me3 peaks with increased (upper panel) or decreased (lower panel) signal in T-PLL. There are 124 and 129 peaks (two middle panels) that show increased signals in P2/P4/P5 and P3/P1/P6, respectively. Differential peaks were identified using the diffbind package, using merged peaks present in at least two of the nine samples. Discriminatory peaks identified in k-means clustering were shown in the heatmap. K-means clustering and heatmap were performed using the number of reads per kb per 10 M, after input subtraction, log2 transformation and quantile normalization. (**c**) TF motifs enriched in the differential H3K27ac peaks gaining (upper panel) or losing occupancy (lower panel) in T-PLL. (**d**) TF motifs enriched in the differential H3K4me3 peaks gaining (upper panel) or losing occupancy (lower panel) in T-PLL. Top 50 motifs with the lowest p values from Homer analysis (http://homer.ucsd.edu/homer/) were displayed. Basic leucine zipper TF family members BATF, JUNB and AP-1 were shown in blue.

**Figure 4:**
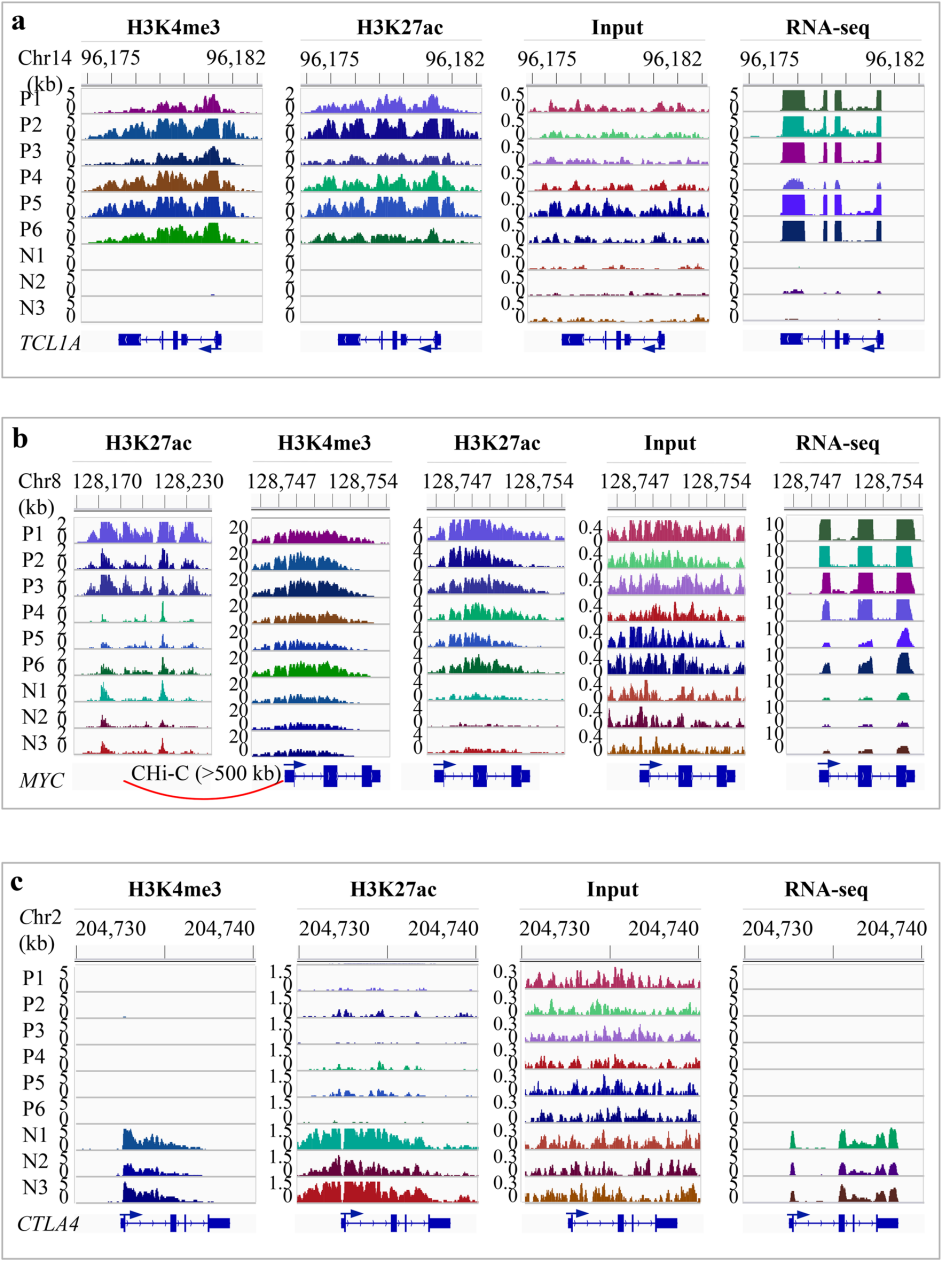
IGV snapshots showing the changes of histone marks and gene expression in T-PLL. (**a**) Increased expression of *TCL1A* and the associated gains of H3K4me3 and H3K27ac. *TCL1A* was actively expressed in T-PLL but not in normal. All T-PLL cases gained both H3K4me3 and H3K27ac peaks (~ 6-kb) that covered the promoter and gene body. Based on the read density in the inputs, there are copy number gains in two T-PLL (P1 and P5). (**b**) Increased expression of *MYC* and the associated increase of H3K4me3 and H3K27ac. *MYC* expression was up-regulated in T-PLL. There was an overall increase in H3K4me3 and H3K27ac occupancy over an ~ 6 kb region that covers the promoter and gene body. The signal level in the inputs suggested a copy number gain in P1, P5 and P6. Promoter capture Hi-C data from 17 blood cell types suggested that an ~ 50-kb enhancer located > 500 kb upstream interacted with *MYC* promoter in total B, naïve B, fetal thymus and five types of T cells. This enhancer showed a marked increase of H3K27ac in three of the T-PLL (P1-P3) compared to the normal. (**c**) Markedly reduced expression of *CTLA4* and the associated loss of H3K4me3 and H3K27ac. The signal in the inputs suggested no copy number alteration within *CTLA4* in T-PLL*.* N1-N3, normal; P1-P6, T-PLL. Y-axis indicates the number of reads per 200-bp (for ChIP-seq and input) or per base pair (for RNA-seq).

Specifically, for H3K27ac we focused on distal peaks, defined as those that are ≥ 2.5 kb away from gene TSS; while for H3K4me3, we focused on proximal peaks in the promoter regions, defined as TSS ± 2.0 kb. Using the top 20,000 most variable (out of 52,248) distal H3K27ac peaks that are present in two or more samples, unsupervised clustering identified a preferential loss of active enhancers in T-PLL (Fig. 2a). Similar results were obtained with the top 10,000 and 30,000 distal H3K27ac peaks (Supplemental Fig. 5a,b). There are 16,629 proximal H3K4me3 peaks within TSS ± 2.0 kb. A similar pattern was identified for the 10,000 most variable H3K4me3 peaks (Fig. 2b). Therefore, there is a genome-wide change of regulatory landscape in T-PLL. As expected, we observed obvious heterogeneity in the occupancy of both H3K4me3 and H3K27ac, showing two sub-groups with P2, P4 and P5 belonging to one and the other three forming the second one (Fig. 2a,b and Supplemental Fig. 5a,b).

We next used the DiffBind package to identify the changes of H3K4me3 and H3K27ac between the four T-PLL (P1, P3 and P5-P6) and three normal that also had total RNA sequencing data. The analysis identified differential occupancy for 15.1% (9362) of the 62,016 H3K27ac peaks and 10.5% (3935) of the 37,306 H3K4me3 peaks (Table 3). As revealed by unsupervised clustering (Fig. 2a,b), there is a strong tendency of losing both marks in T-PLL compared to normal. To understand whether differential peaks are associated with the change of gene expression, we assigned them to the nearest genes and checked the expression changes of those genes. Indeed, for both marks, peaks that showed increased occupancy in T-PLL were preferentially associated with up-regulated in T-PLL, compared to non-differential peaks, and vice versa (enrichment: 3.8–19.2, chi-square test *p* < 2.2e−16) (Table 3). To correlate the changes of gene expression with those of proximal occupancy of H3K4me3 and H3K27ac, for the 865 down- and 807 up-regulated genes identified by total RNA sequencing, we also plotted the average signal profiles of H3K4me3 and H3K27ac over the TSS ± 2 kb (Fig. 2c,d). As expected, overall, the down-regulated genes were associated with decreased H3K4me3 and H3K27ac, and vice versa; the correlated changes were more obvious for the up-regulated genes (Fig. 2d).

To understand which TFs might be involved in the perturbation of T-PLL epigenome, we first used diffbind to identify differential H3K4me3 and H3K27ac peaks on chr1-22 between the six T-PLL and three normal, followed by k-means clustering of the differential peaks. The analysis identified 2445 (1728 + 717) of the 2456 H3K27ac peaks with increased signal in T-PLL and all 5587 peaks with decreased signal as discriminatory peaks (Fig. 3a). To a less extent, 80% (647 + 124 + 129 = 900) of the 1128 H3K4me3 peaks with increased signal in T-PLL and 90% (2658) of the 2971 peaks with decreased signal were retained (Fig. 3b). The other differential peaks showed less difference between T-PLL and normal. We then used the Homer package to identify known TF motifs that are enriched in these discriminatory peaks (Fig. 3c,d). The differential H3K27ac peaks (Fig. 3c), as well as H3K4me3 peaks gained in T-PLL (upper panel in Fig. 3d) are enriched for binding motifs of E-twenty-six (ETS) TFs, such as ERG, ETS1, and ELF4. On the other hand, differential H3K4me3 peaks lost in T-PLL (lower panel in Fig. 3d) are particularly enriched for Interferon regulatory factor (IRF) TF motifs. Finally, for both marks, the differential peaks lost in T-PLL are enriched for basic-region leucine zipper (bZIP) TF motifs including BATF, AP-1 and JUNB (Fig. 3c,d, in blue) that are not enriched in peaks gained in T-PLL. In fact, many of them are immune related TFs, including lineage-specific TFs such as SCL, PU.1, and TCF12^47,48^. For example, BATF, JUNB, and PU.1 interact with IRF4, which plays critical roles in lymphoid development and immune response^49^.

To reveal the extent by which CNA may contribute to epigenomic change, we identified CNAs in individual T-PLL cases based on sequence coverage in 100-kb windows along chr1-22. At the cutoff of absolute z-score >  = 3, there are on average 0.34% and 0.37% of the windows that showed CN gain and loss, respectively. However, peaks are > sevenfold over-represented in the windows with CN gain compared to those with CN loss. Specifically, for the consensus peaks shared by at least two T-PLL, 0.78–5.20% of the H3K27ac peaks and similar proportions of the H3K4me3 peaks (0.66–4.82%) fall in windows with CN gain, versus <  = 0.6% in windows with CN loss (Supplemental Table 4). This bias is unlikely due to peak calling, as a similar trend was also observed for additional two consensus peak sets of each mark, i.e., those present in >  = 2 T-PLL and >  = 2 normal, as well as those present in >  = 2 normal but absent from all T-PLL. In these additional peak sets, < 0.5% of peaks overlap windows with CN loss, changing little (< 1%) at a less stringent cutoff of z-score <  = − 2. The results indicate that regions with CN loss are less likely to be enriched with H3K4me3 and H3K27ac modifications. Finally, we investigated the overlap of differential peaks with windows carrying CNAs (Supplemental Table 4). Intersecting both marks with windows carrying CN gain revealed overlap with about 3–4% the peaks with increased signal in T-PLL, versus with 0.6% (H3K27ac) and 1.94% (H3K4me3) of the peaks with decreased signal in T-PLL. Thus, even for peaks falling within windows with CN gain, some showed reduced histone modification levels in T-PLL instead. On the other hand, similar to the whole peak set, differential peaks are much less associated with windows with CN loss. It is possible that, with a large (100-kb) window size and a modest coverage, small CNAs overlapping differential peaks may not have been identified, and that this method may be less effective in the detection of CN loss compared to that of CN gain. Nevertheless, CNAs were found to contribute to the changes of H3K4me3 and H3K27ac in a small portion of peaks, including those associated with key genes (such as *MYC* and *AGO2*) described below.

### Changes in chromatin states of key dysregulated genes

As revealed by a recent study^5^ and our RNA-seq data described above, T-PLL is characterized by the dysregulation of several oncogenes, such as *TCL1A*, *MYC* and *EZH2*, as well as genes implicated in DNA damage repair (e.g., *ATM*) and T-cell receptor regulation (such as *CTLA4* and SLAM family members). Below we focused on these key genes. Specifically, we aimed to illustrate the epigenetic state in the regulatory regions of these genes and to understand how epigenetic alterations have modulated the transcriptional outcome.

The hallmark of genetic alterations in T-PLL includes t(14;14)(q11;q32) and inv(14)(q11q32) that involve the proto-oncogene *TCL1A*. *TCL1A* activates the serine/threonine kinase AKT that is implicated in cell survival regulation, as well as the NF-κB signaling pathway that plays key roles in the initiation and promotion of cancer^6,50^. Increased *TCL1A* expression is associated with less favorable clinical outcome in chronic lymphocytic leukemia^6,51^. *TCL1A* was significantly up-regulated in T-PLL compared to normal (log_2_FC = 5.60 and *p* = 3.30e−08 in total RNA-seq; log_2_FC = 7.87 and *p* = 1.08e−18 in mRNA-seq) (Fig. 4a, right panel). All six T-PLL cases demonstrated a gain of H3K4me3 and H3K27ac over its promoter and gene-body (Fig. 4a), which, we believe, contributes to *TCL1A* activation in addition to the known rearrangement of *TCL1A* locus. Based on the signal level in the corresponding input, at least two of the T-PLL samples (P1 and P5) showed copy number gains in *TCL1A*, which may have also resulted in increase of *TCL1A* expression, as well as of H3K4me3 and H3K27ac occupancy.

To examine whether this ~ 6-kb H3K27ac peak exists in other tissue and cell types, we first checked the 98 human reference epigenomes that have H3K27ac data^28^. H3K27ac enrichment was detected only in a primary B-cell sample and in GM12878, which is a B cell-derived lymphoblastoid cell line. Based on other publicly available H3K27ac ChIP-seq data we collected, this broad peak was identified in four of the five normal B-cell samples, in patient biopsies from all seven mantle cell lymphoma, three small lymphocytic lymphoma and four of the seven high-grade B-cell lymphoma subjects (Supplemental Fig. 6), but not in any of the 10 adult T-cell leukemia/lymphoma (ATL) patients^35^. In addition, based on the catalog of super-enhancers from 86 cell and tissue types^36^, this enhancer is part of a B-cell specific super-enhancer. These results suggest a possibility that requisition of this cell-type restricted enhancer activates the oncogenic expression of *TCL1A* in T-PLL*.*

Oncogenic activation of *MYC* in T cell leukemia^52^ and B cell lymphoma^33^ involves interaction between its promoter with subtype specific enhancers. T-PLL is also characterized by the activation of *MYC*^5^. We detected up-regulation of *MYC* expression (log_2_FC = 2.83 and *p* = 5.13e−03 in total RNA-seq; log_2_FC = 3.37 and *p* = 9.49e−06 in mRNA-seq). To support a role for epigenetic mechanism in *MYC* activation, ChIP-seq data showed an overall increase of H3K4me3 and H3K27ac occupancy over an ~ 6 kb region in all T-PLL cases that covers *MYC* promoter and gene body (Fig. 4b). The signal in the inputs suggested copy number gains in P1, P5 and P6. To identify the enhancer that potentially interacts with the *MYC* promoter, we used the promoter CHi-C data from 17 blood cell types^37^. The analysis identified an ~ 50-kb enhancer located over 500 kb upstream that interacted with the *MYC* promoter in total B, naïve B, fetal thymus and five of the six types of T cells (Fig. 4b). This broad enhancer showed marked increase of H3K27ac in three of the T-PLL cases (P1-P3). Thus, in these three cases, the up-regulation of *MYC* expression appears to be driven by the increased H3K27ac in the enhancer together with increased H3K4me3 and H3K27ac in the promoter. For the other three cases (P4-P6), the increased *MYC* expression is associated with a gain of H3K27ac in the promoter in all three, and a gain of H3K4me3 in P6.

*EZH2* is an oncogene encoding a histone methyltransferase that deposits H3K27me3, a histone mark associated with transcriptional repression^53^. *EZH2* is highly expressed in several B-cell lymphomas^53^. We found that *EZH2* was up-regulated in four of the T-PLL samples (P2-P4 and P6) (Supplemental Fig. 7). Three of the four (P3, P4, and P6) also showed increased occupancy of both H3K4me3 and H3K27ac in the promoter (Supplemental Fig. 7), with the exception of P2 that had a copy-number loss indicated by the lower signal in the input. Overall, the elevated *EZH2* expression in T-PLL is associated with increased H3K4me3 and H3K27ac in its promoter.

In the argonaute (AGO) family, only *AGO2* has the catalytic activity and plays an essential role in small RNA-guided gene silencing^54^. Often up-regulated in cancer, *AGO2* is involved in chromatin remodeling and alternative splicing^5^. *AGO2* was up-regulated in P1 and P2 (Supplemental Fig. 8). All the six T-PLL samples showed increased H3K4me3 and H3K27ac in its promoter (Supplemental Fig. 8). The signal level in inputs suggested that four (P1, P3, P5 and P6) had copy-number gains, which likely have contributed to the increased H3K4me3 and H3K27ac in these cases. For P2 that showed no copy number alteration, its up-regulation may be directly due to the increased H3K4me3 and H3K27ac.

On the other hand, we also checked the chromatin states for several T-cell receptor regulators and *ATM*. As a major negative regulator of T-cell response, *CTLA4* was markedly down-regulated in T-PLL (log_2_FC = − 6.96 and *p* = 8.46e−14 in total RNA-seq; log_2_FC = − 6.27 and *p* = 1.08e−16 in mRNA-seq), which was associated with an obvious reduction of H3K4me3 and H3K27ac (Fig. 4c). Input signal indicated no CNV in any of the T-PLL cases. The SLAM family members also act as the negative regulators of T-cell response. Of the nine members, seven reside in a 378-kb region on chromosome 1 (Supplemental Fig. 9). Of the seven, *CD84* (also known as *SLAMF5,* log_2_FC = − 3.69 and *p* = 2.22e−05 in total RNA-seq; log_2_FC = − 4.47 and *p* = 2.85e−10 in mRNA-seq), *SLAMF7* (log_2_FC = − 3.12 and *p* = 4.44e−04 in total RNA-seq; log_2_FC = − 3.78 and *p* = 7.42e−08 in mRNA-seq) and *CD244* (*SLAMF4,* log_2_FC = − 2.17 and *p* = 2.26e−02 in total RNA-seq; log_2_FC = − 2.34 and *p* = 1.16e−03 in mRNA-seq) were down-regulated in all T-PLL cases. Accordingly, these three genes showed lower levels of H3K4me3 in their promoters compared to the normal. In addition, *SLAMF6* was down-regulated in P2, P4 and P5, showing lower H3K4me3 in promoter in P4 and P5; *SLAMF1* was down-regulated in P1, P3 and P4, with lower H3K4me3 in promoter in the first two. Clearly, for genes involved in the regulation of T-cell response, alterations of chromatin state in the promoters are linked to their dysregulation.

In B-cell chronic lymphocytic leukemia, ATM was found to physically interact with TCL1A protein, and this association activates the NF-κB pathway in the regulation of apoptosis^6,55^. *ATM* aberration is viewed as a key initiating lesion in T-PLL^5^. *ATM* showed lower H3K4me3 occupancy in the promoter in all T-PLL cases, with down-regulation of expression in four of them (except in P1 and P5) (Supplemental Fig. 10). Based on the input signal within *ATM*, there was a copy-number loss in five T-PLL cases (P2-P6), which may have led to the observed changes in epigenetic state and expression. The result suggested a link between the reduction of H3K4me3 in promoter and *ATM* down-regulation. Collectively, our analysis demonstrated a central role for epigenetic mechanisms in the dysregulation of key oncogenes, and genes with roles in DNA damage repair and T-cell receptor regulation in T-PLL.

## Discussion

T-PLL was best characterized by oncogenic expression of *TCL1A* and dysfunction of *ATM*^5^. Our analysis validated the previous genomic findings of dysregulated genes and further suggested key roles for epigenetic mechanisms in transcriptional dysregulation. We identified genome-wide alterations of chromatin states at both promoters and active enhancers in T-PLL, which likely involves immune related TFs that are highly enriched in differential peaks. The results supported the epigenetic regulation of oncogene activation and repression of genes involved in DNA damage and T-cell responses. Importantly, we provided evidence for the gained enhancers in T-PLL that contribute to the overexpression of *TCL1A* and *MYC*. We believe the same mechanism underlies their activation in several other hematological malignancies. For example, a super-enhancer within *TCL1A* was identified in high-grade B-cell lymphoma, mantle cell lymphoma and small lymphocytic lymphoma^33,34^. Accordingly, *TCL1A* showed overexpression in these B-cell lymphomas^56,57^. In B-cell chronic lymphocytic leukemia, *TCL1A* up-regulation was also associated with the gained super-enhancer^58^ and with DNA hypomethylation in its promoter^55^. Additionally, in many other cancers including T cell leukemia, chronic myeloid leukemia and multiple myeloma, *MYC* expression was linked to the super-enhancers acquired in tumor cells^36^. Thus, this study has enhanced our understanding of epigenetic regulation of gene expression in T-PLL.

Our study is limited by a small sample size because T-PLL is a rare mature T cell leukemia. Nevertheless, we revealed an obvious heterogeneity in gene regulatory landscape within T-PLL, with P2, P4 and P5 in one group and the other three in the second group. We also identified marked differences in gene expression across T-PLL cases. Further study using a larger collection of samples is needed to identify the epigenetic and transcriptional programs associated with disease progression.

Our analysis supported roles for epigenetic mechanisms in gene dysregulation in T-PLL. Genetic alterations were found to contribute to transcriptional abnormality in T-PLL^5^. Based on input signals, two to four of the T-PLL cases had copy-number gains in *AGO2*, *MYC* and *TCL1A* that were accompanied by increased H3K27ac and H3K4me3, while copy-number loss was observed in *ATM* in five T-PLL cases (P2-P6) that was accompanied by decreased H3K4me3. Complementarily, CNA analysis with 100-kb windows identified CN gains covering *AGO2* and *MYC*, and also *TCL1A* at a less stringent cutoff of z = 2. For these four genes, the CNVs inferred from input signal spanned both gene-body and promoter. In fact, copy-number gain in *AGO2* and *MYC* and copy-number loss in *ATM* are the major genomic aberrations in T-PLL ^5^. Genomic rearrangements in regulatory regions represent an important mechanism for oncogenic activation. For example, in acute myeloid leukemia^59^ and acute lymphoblastic leukemia^60^, the distal super-enhancers, 1.7 and 1.3 Mb away from the *MYC* promoter, respectively, were located in regions of focal amplification. Integrative analysis of genetic variation, epigenetic profiles with gene expression will help decipher the roles of interplay between genetic and epigenetic factors in shaping the transcriptional program in T-PLL.

In summary, the analyses have identified the transcriptional changes for genes enriched in immune and inflammatory responses, as well as for those in WNT signaling network. Using H3K4me3 and H3K27ac ChIP-seq data, we revealed genome-wide alterations of chromatin states at both promoters and active enhancers in T-PLL. Integrative analysis with RNA-seq data suggests the changes of regulatory regions as a major mechanism underlying transcriptional reprogramming. Importantly, epigenetic changes in regulatory regions are evident at key oncogenes and genes involved in DNA damage response and T-cell receptor regulation, which are tightly linked to their transcriptional dysregulation, the core lesions of T-PLL. Collectively, this study enhances our understanding of the roles of epigenetic mechanisms in T-PLL pathogenesis.

## Supplementary Information

Supplementary Information

## Data Availability

The processed RNA-seq and ChIP-seq data generated in this study are available in GEO under the accession GSE143374.
