# Supplementary material for: Epigenetic alteration contributes to the transcriptional reprogramming in T-cell prolymphocytic leukemia
Source: Sci Rep. 2021 Apr 15;11:8318. doi: 10.1038/s41598-021-87890-9 (PMC8050249; doi:10.1038/s41598-021-87890-9)
Supplement: Supplementary file 1 — Supplementary Information [file 41598_2021_87890_MOESM1_ESM.pdf]

Supplemental Figures and Tables

Epigenetic alteration contributes to the transcriptional reprogramming in T-cell prolymphocytic leukemia

Shulan Tian[1], Henan Zhang[2], Pan Zhang[3+], Michael Kalmbach[3], Jeong-Heon Lee[4], Tamas Ordog[5], Paul J. Hampel[6], Timothy G. Call[6], Thomas E. Witzig[6], Neil E. Kay[6], Eric W. Klee[1], Susan L. Slager[1], Huihuang Yan[1] & Wei Ding[6]

[1]Division of Biomedical Statistics and Informatics, Department of Health Sciences Research, Mayo Clinic, Rochester, MN, USA
[2]Department of Urology, Mayo Clinic, Rochester, MN, USA
[3]Division of Information Management and Analytics, Department of Information Technology, Mayo Clinic, Rochester, MN, USA
[4]Division of Experimental Pathology and Laboratory Medicine, Department of Laboratory Medicine and Pathology, Mayo Clinic, Rochester, MN, USA
[5]Department of Physiology and Biomedical Engineering, Mayo Clinic, Rochester, MN, USA
[6]Division of Hematology, Mayo Clinic, Rochester, MN, USA

[+]Current address: Illinois Informatics Institute, University of Illinois at Urbana-Champaign, Urbana, IL, USA

Supplemental Fig. 1 Gene expression changes in T-PLL detected by mRNA sequencing. (**a**) Unsupervised clustering of expression level from top 10 000 most variable genes. N4-N7, normal individuals; P2, P4, and P7-P9, T-PLL patients. Z score was calculated from $\log_2$(RPKM+0.1). (**b**) Protein-coding genes differentially expressed between T-PLL and normal. The 2 364 (1466+898) differentially expressed genes were identified using edgeR at the cutoff of 5% FDR and 3-fold change. Y-axis, fold-change at the $\log_2$ scale; x-axis, sum of the normalized read count per M from the control (normal) and T-PLL group. See Fig. 1 legend for additional information.

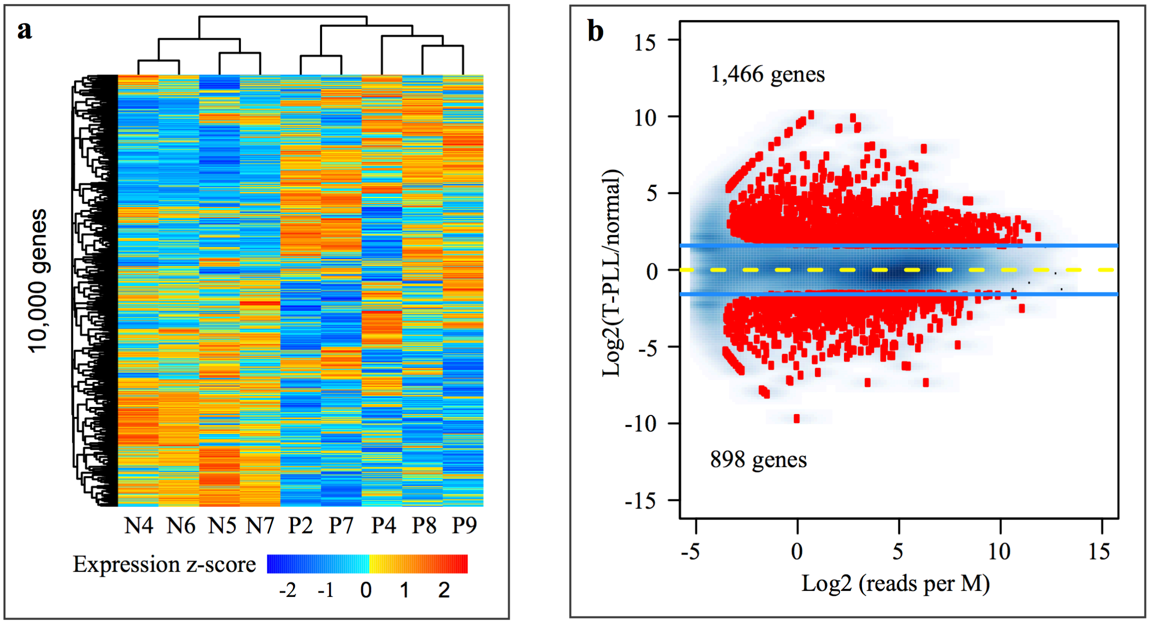

Supplemental Fig. 2 Sequencing depth and expression levels of DEGs. (**a**) Total RNA and mRNA sequencing reads mapped to all genes and to protein-coding genes. (**b**) Boxplot showing expression level of protein-coding genes up-regulated in T-PLL revealed by both (left panel), by mRNA sequencing only (middle panel), and by total RNA sequencing only (right panel). The differentially expressed genes (DEGs) were identified using edgeR at the cutoff of FDR <= 5% and fold change >= 3. Y-axis represents the gene maximum RPKM value (after log2 transformation with an offset of 0.1) across both T-PLL and normal, calculated separately for mRNA and total RNA sequencing data. P value was calculated using one-sided Mann–Whitney U test (paired=TRUE). Total RNA sequencing was performed for 3 normal (N1-N3) and 4 T-PLL (P1, P3, P5 and P6) and mRNA sequencing for 4 normal (N4-N7) and 5 T-PLL (P2, P4, and P7-P9). See Table 1 for details about sample information.

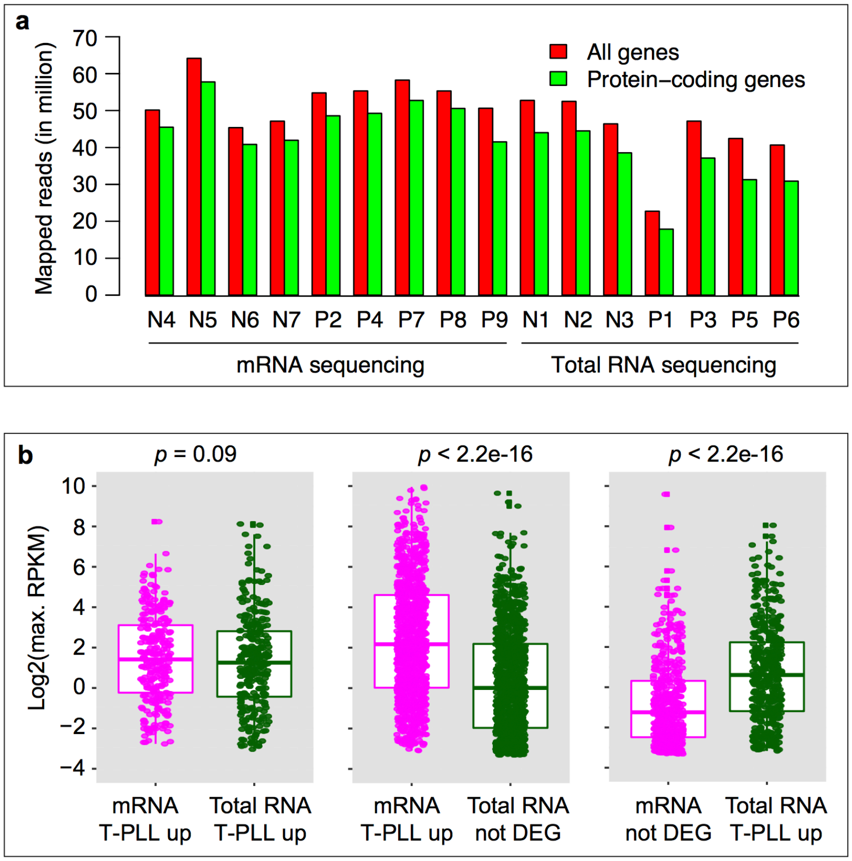

Supplemental Fig. 3 Pairwise Pearson correlation coefficient for H3K4me3 (**a**) and H3K27ac (**b**). Only merged peaks from chr1-22 that are present in at least two samples were used. Raw read counts in merged peaks were input-subtracted, normalized to 10M mapped reads, log2 transformed and quantile normalized. N1-N3, normal individuals; P1-P6, T-PLL patients.

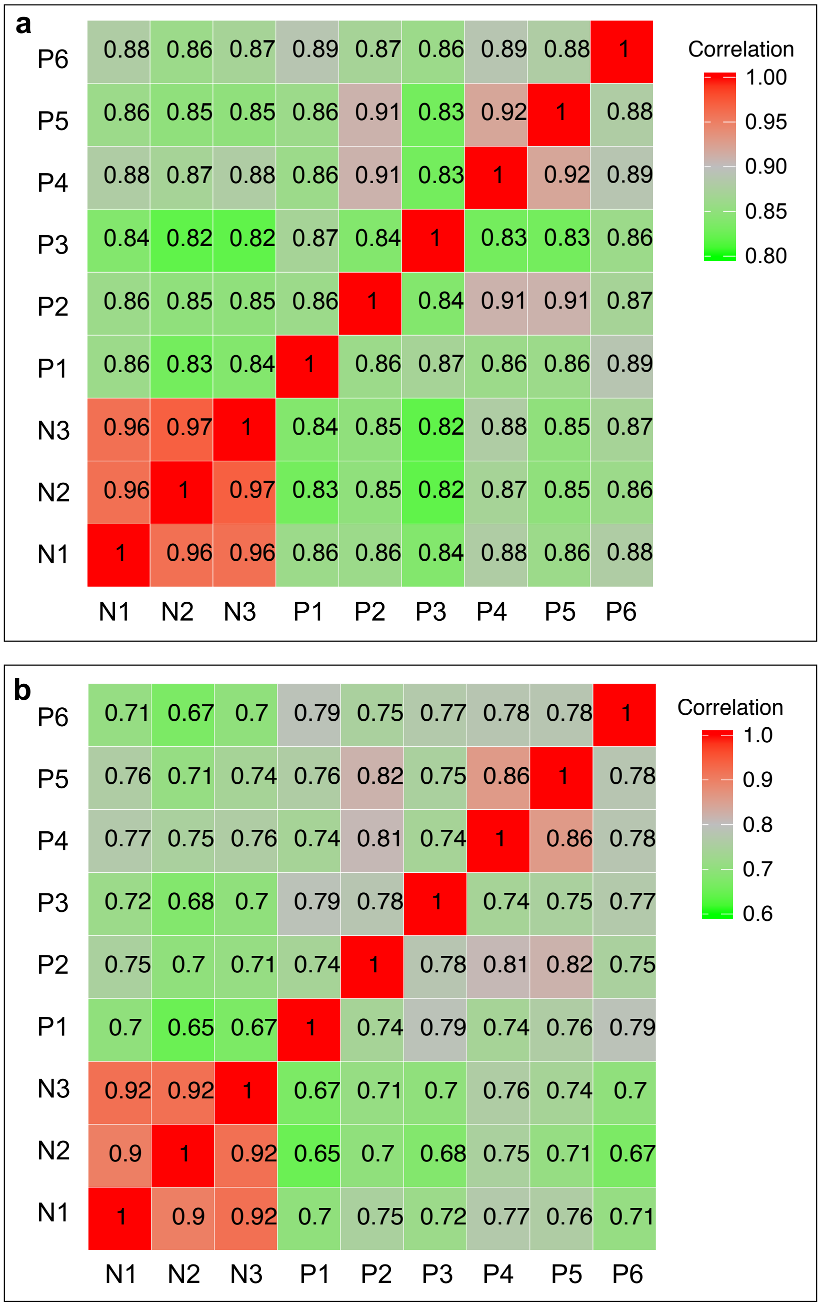

Supplemental Fig. 4 T-PLL H3K27ac summary and tumor suppressor gene expression. (**a**). Summary of H3K27ac ChIP-seq in 6 T-PLL. [a]HDACi, romidepsin treatment; [b]FC (IP/INPUT fold change), fraction of uniquely mapped reads within peaks in IP / fraction of uniquely mapped reads within peak corresponding regions in INPUT, with duplicate removed; [c]PCC, Pearson correlation coefficient between each T-PLL and the other 5 T-PLL (see Supplemental Fig. 3 for details). (**b**) Signal level of H3K27ac peaks present in all 6 T-PLL (left) and in at least 2 T-PLL (right). RP10M, IP reads per 10M uniquely mapped reads after subtraction of reads from input. Dashed line represents the median $\log_2$(RP10M) of sample P1. (**c**) Expression level of tumor suppressor genes based on total RNA sequencing. Tumor suppressor genes were from https://bioinfo.uth.edu/TSGene/, where only protein-coding genes were used. N1-N3: normal; P1, P3, P5, P6: T-PLL.

a

Sample	HDACi[a]	Usable reads	No. peak	FC[b]	PCC[c]
P1	Yes	27,358,173	85,085	6.75	0.74-0.79
P2	No	29,865,798	86,364	8.14	0.74-0.82
P3	No	24,393,414	79,491	6.42	0.75-0.79
P4	No	21,651,800	87,942	8.02	0.74-0.86
P5	No	29,593,013	90,794	5.6	0.75-0.86
P6	No	19,815,677	69,842	5.3	0.75-0.79

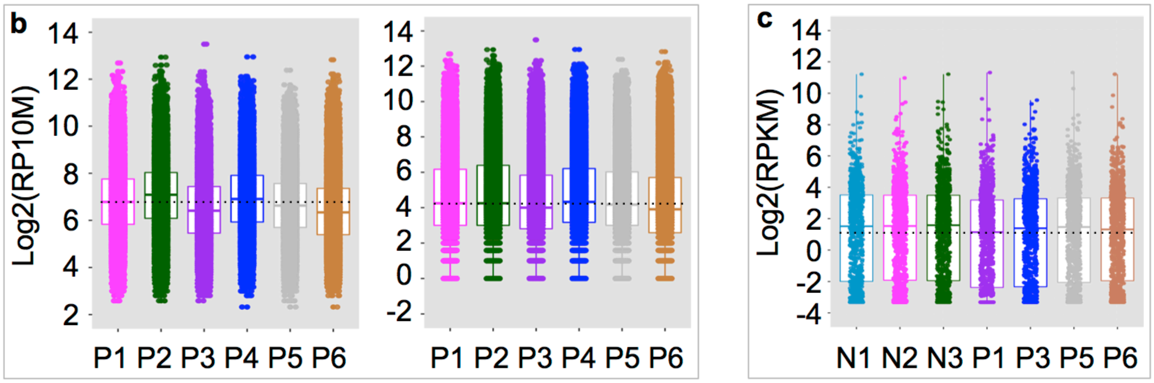

Supplemental Fig. 5 Global alteration of distal H3K27ac peaks in T-PLL. (**a**) Unsupervised clustering of top 10,000 H3K27ac peaks. (**b**) Unsupervised clustering of top 30,000 H3K27ac peaks. See Fig. 2 legend for details.

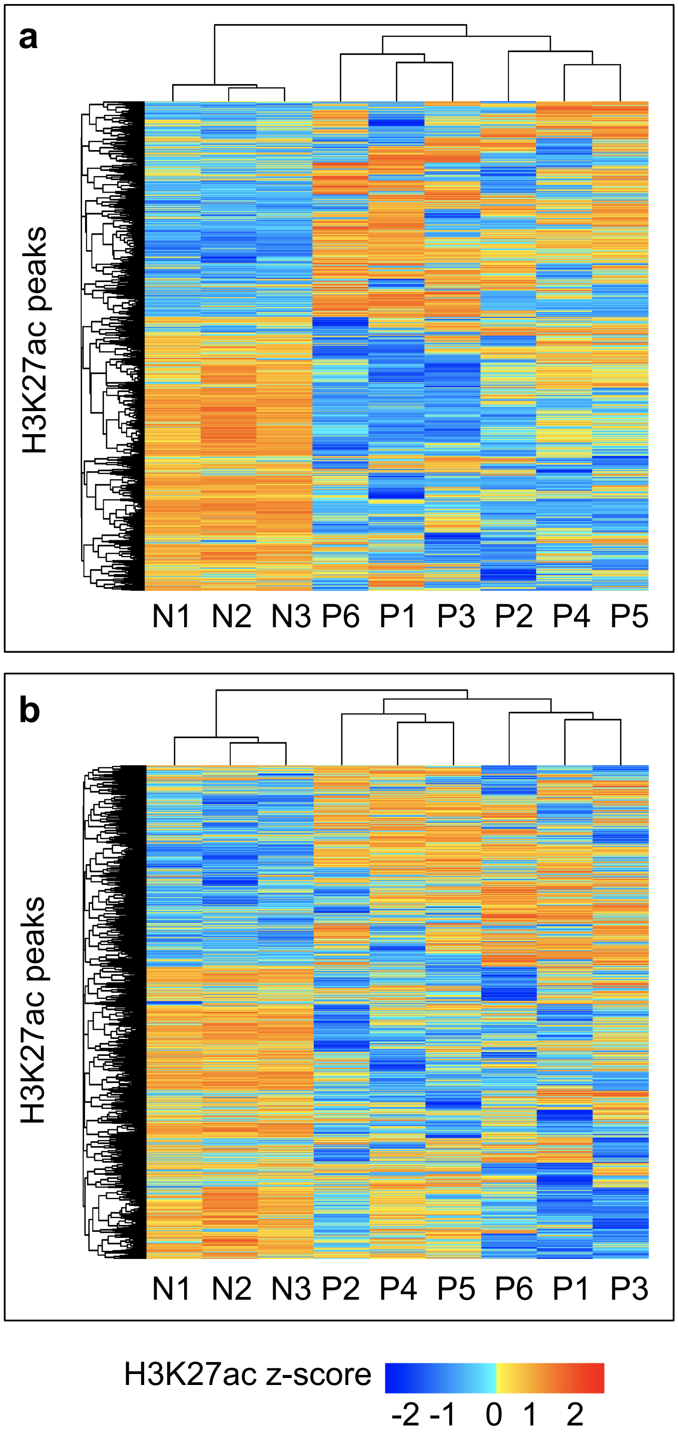

Supplemental Fig. 6 Gained enhancer in *TCL1A* in T-PLL. In the catalog of super-enhancers identified from 86 cell and tissue types, only B cells had the super-enhancer. This super-enhancer was also present in four of the seven high-grade B-cell lymphoma (HGB), all seven mantle cell lymphoma (MCL) and all the three small lymphocytic lymphoma (SLL) patient samples. The raw H3K27ac ChIP-seq reads for four of the five normal B-cell samples were downloaded from GEO under the accession GSM998996, GSM1027287, GSM998997 and GSM2386722. Alignments for the remaining B-cell sample were from Roadmap Epigenomics Project (reference epigenome identifier [EID]: E032).

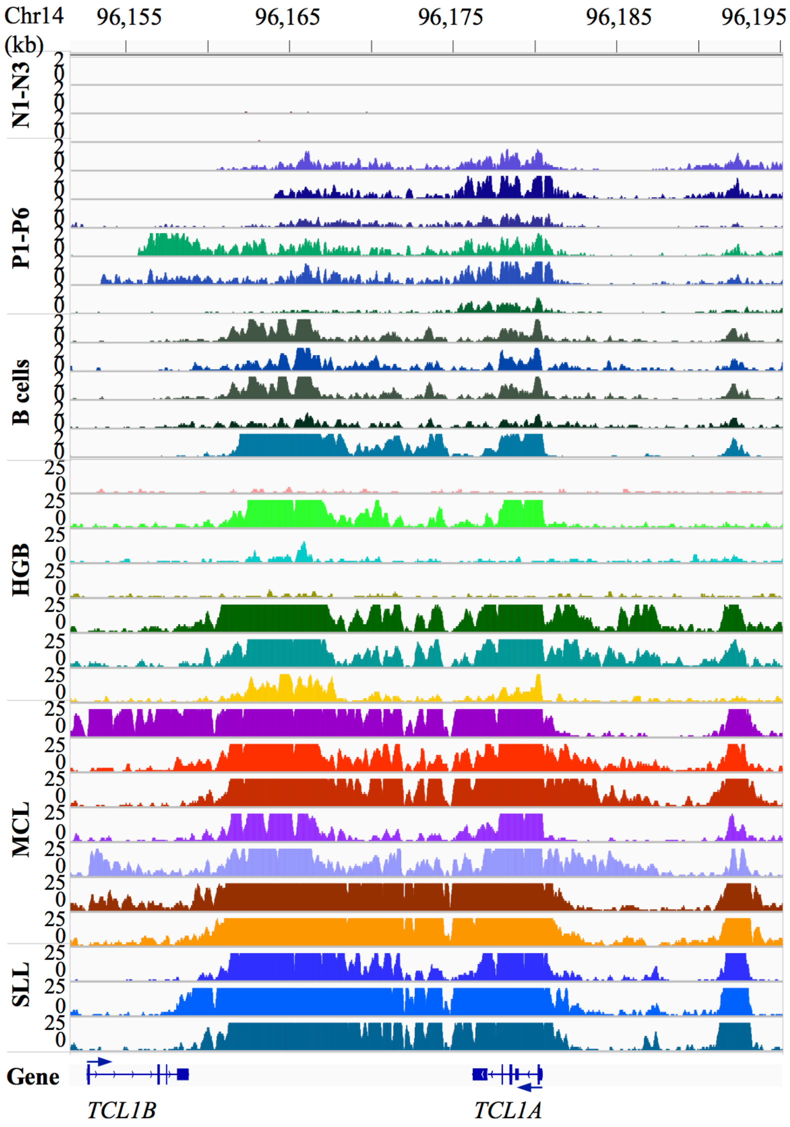

Supplemental Fig. 7 Overall increase of H3K4me3 and H3K27ac in the *EZH2* promoter in T-PLL. *EZH2* was up-regulated in four of the T-PLL cases (P2-P4 and P6). Three of them (P3, P4, and P6) showed increased H3K4me3 and H3K27ac in the promoter. Based on the signal in input, there was a copy-number loss in P2.

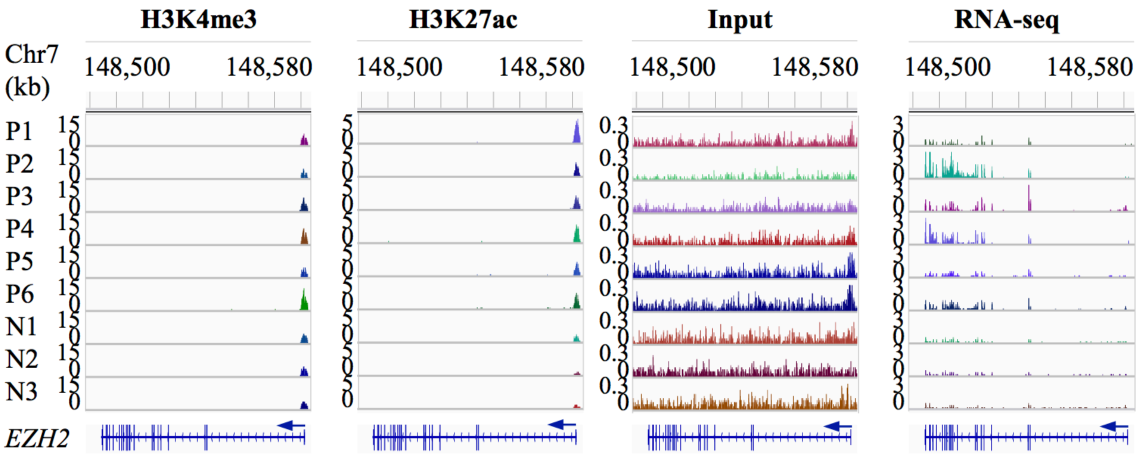

Supplemental Fig. 8 Increased H3K4me3 and H3K27ac in the *AGO2* promoter in T-PLL. All six T-PLL showed increased occupancy of both H3K4me3 and H3K27ac in the *AGO2* promoter. *AGO2* showed elevated expression in two of the T-PLL cases (P1 and P2). Based on input signal, four of the T-PLL (P1, P3, P5 and P6) had copy number gains.

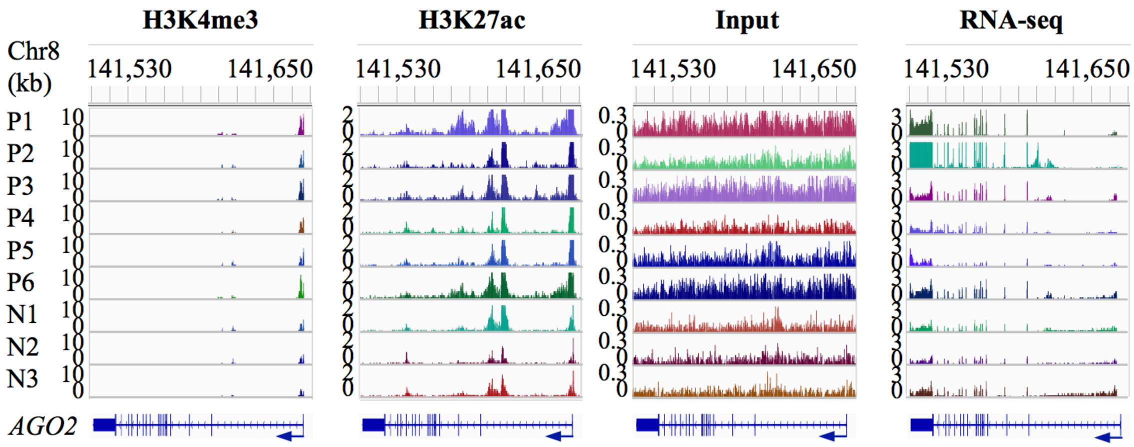

Supplemental Fig. 9 Expression and epigenetic changes in the SLAM family genes. There are seven SLAM family genes in a 378-kb region on chromosome 1. The expression of *CD84*, *SLAMF7* and *CD244* was down-regulated in all T-PLL cases. Accordingly, these genes showed lower levels of H3K4me3 in their promoters. Another two genes, *SLAMF6* and *SLAMF1*, were down-regulated in three T-PLL cases. Of the three, P4 and P5 had lower H3K4me3 in *SLAMF6* promoter and P1 and P3 had lower H3K4me3 in *SLAMF1* promoter.

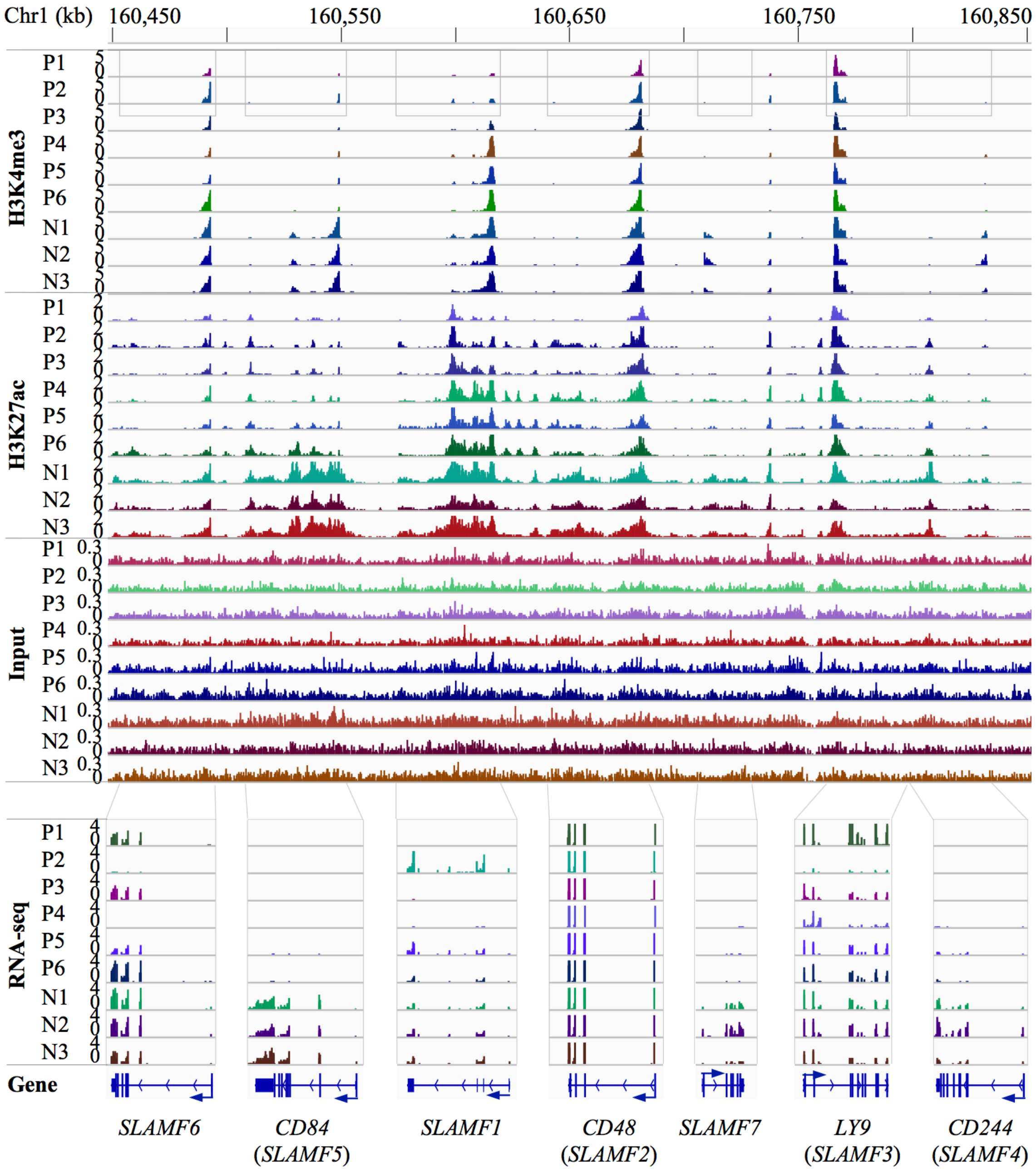

Supplemental Fig. 10 Reduced H3K4me3 in the *ATM* promoter in T-PLL. All six T-PLL cases showed lower H3K4me3 occupancy in the promoter. *ATM* was down-regulated in four of the T-PLL cases (P2-P4 and P6) that also showed a copy number loss based on input signal.

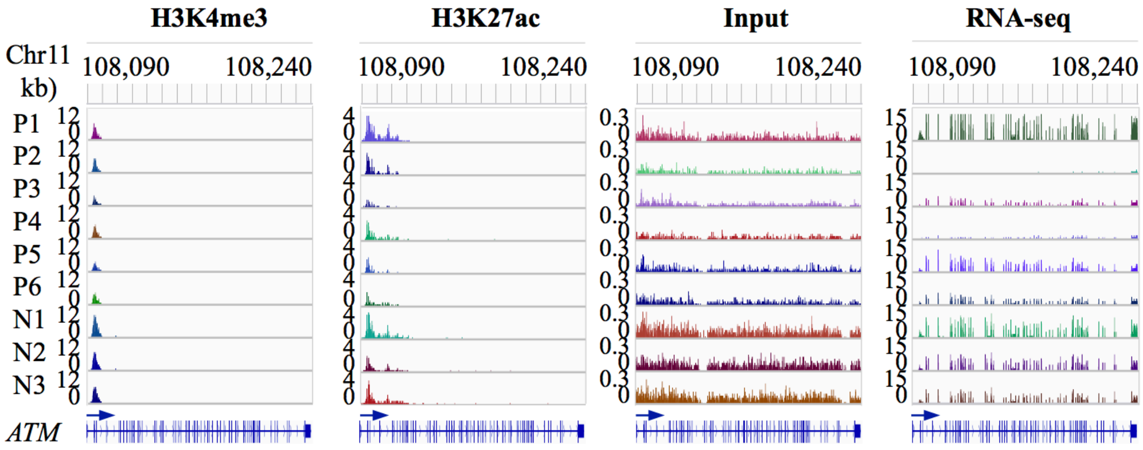

Supplemental Table 1. Key pathways and GO terms associated with down-regulated genes in T-PLL

**Name**	**No. gene**	**Q value**	**Source**
Immune System	172	4.50E-12	Reactome
Chemokine receptors bind chemokines	20	4.50E-12	Reactome
Peptide ligand-binding receptors	32	4.97E-08	Reactome
Immunoregulatory interactions between a Lymphoid and a non-Lymphoid cell	26	8.70E-08	Reactome
Neutrophil degranulation	51	6.97E-06	Reactome
Signaling by Interleukins	53	1.33E-05	Reactome
Hemostasis	60	2.07E-05	Reactome
Innate Immune System	98	5.19E-05	Reactome
Cytokine Signaling in Immune system	64	1.75E-04	Reactome
Adaptive Immune System	59	4.10E-02	Reactome
Immune response (GO:0006955)	203	1.50E-26	GO
Immune system process (GO:0002376)	249	9.80E-25	GO
Cell activation (GO:0001775)	147	3.57E-22	GO
Regulation of immune system process (GO:0002682)	150	7.90E-19	GO
Defense response (GO:0006952)	154	3.03E-17	GO
Leukocyte activation (GO:0045321)	121	3.28E-15	GO
Positive regulation of immune system process (GO:0002684)	109	2.65E-14	GO
Regulation of immune response (GO:0050776)	103	6.52E-13	GO
Leukocyte mediated immunity (GO:0002443)	89	1.54E-12	GO
Chemokine-mediated signaling pathway (GO:0070098)	25	1.71E-12	GO

Gene expression was quantified by mRNA sequencing for four normal (N4-N7) and five T-PLL (P2, P4, and P7-P9).

Supplemental Table 2. Key pathways and GO terms associated with genes up-regulated in T-PLL

Name	No. gene	Q value	Source
Erythrocytes take up oxygen and release carbon dioxide	6	2.36E-04	Reactome
O2/CO2 exchange in erythrocytes	6	1.33E-03	Reactome
Extracellular matrix organization	29	1.33E-03	Reactome
MET activates PTK2 signaling	8	5.38E-03	Reactome
Degradation of the extracellular matrix	16	1.95E-02	Reactome
MET promotes cell motility	8	2.89E-02	Reactome
EPHA-mediated growth cone collapse	7	2.89E-02	Reactome
Cell surface interactions at the vascular wall	15	2.89E-02	Reactome
Alpha-defensins	4	3.63E-02	Reactome
G1/S-Specific Transcription	5	4.12E-02	Reactome
Multicellular organism development (GO:0007275)	269	1.12E-07	GO
System development (GO:0048731)	237	8.94E-07	GO
Developmental process (GO:0032502)	300	6.70E-06	GO
Oxygen carrier activity (GO:0005344)	6	9.58E-06	GO
Oxygen binding (GO:0019825)	9	9.22E-05	GO
Circulatory system development (GO:0072359)	68	1.86E-04	GO
Wnt-protein binding (GO:0017147)	6	1.31E-03	GO
Cell differentiation (GO:0030154)	189	1.88E-03	GO
Regulation of cell proliferation (GO:0042127)	85	4.14E-02	GO
Wnt-activated receptor activity (GO:0042813)	3	4.47E-02	GO

Gene expression was quantified by total RNA sequencing for three normal (N1-N3) and four T-PLL (P1, P3, P5 and P6).

Supplemental Table 3. Key pathways and GO terms associated with up-regulated genes in T-PLL

Name	No. gene	Q value	Source
Axon guidance	67	3.80E-03	Reactome
Signal Transduction	218	8.03E-03	Reactome
Metallothioneins bind metals	6	8.03E-03	Reactome
Neuronal System	43	1.19E-02	Reactome
EPH-ephrin mediated repulsion of cells	12	1.20E-02	Reactome
Extracellular matrix organization	38	1.29E-02	Reactome
EPH-Ephrin signaling	17	1.53E-02	Reactome
Signaling by Interleukins	57	1.94E-02	Reactome
Cytokine Signaling in Immune system	75	2.79E-02	Reactome
Developmental Biology	101	3.04E-02	Reactome
Multicellular organism development (GO:0007275)	536	2.59E-24	GO
Developmental process (GO:0032502)	601	1.71E-23	GO
System development (GO:0048731)	482	6.57E-22	GO
Circulatory system development (GO:0072359)	130	7.51E-10	GO
Cell differentiation (GO:0030154)	404	3.30E-14	GO
Regulation of cell differentiation (GO:0045595)	198	7.81E-11	GO
Wnt-protein binding (GO:0017147)	10	1.63E-03	GO
Wnt-activated receptor activity (GO:0042813)	8	2.49E-03	GO
Transcription regulatory region DNA binding (GO:0044212)	101	1.32E-05	GO
Ephrin receptor activity (GO:0005003)	9	2.07E-04	GO

Gene expression was quantified by mRNA sequencing for four normal (N4-N7) and five T-PLL (P2, P4, and P7-P9).

Supplemental Table 4. Copy number alterations in regulatory regions in T-PLL

Sample	Mark	No. peak*		No. peak[+]		Up in T-PLL§		Down in T-PLL§	
		Gain (%)	Loss (%)	Gain (%)	Loss (%)	Gain (%)	Loss (%)	Gain (%)	Loss (%)
P1	H3K27ac	600 (1.10)	80 (0.15)	680 (0.95)	85 (0.12)	84 (3.42)	6 (0.24)	6 (0.11)	0 (0.00)
P2	H3K27ac	1265 (2.31)	176 (0.32)	1537 (2.15)	203 (0.28)	103 (4.19)	5 (0.20)	39 (0.70)	4 (0.07)
P3	H3K27ac	1905 (3.48)	73 (0.13)	2372 (3.32)	81 (0.11)	142 (5.78)	2 (0.08)	71 (1.27)	0 (0.00)
P4	H3K27ac	425 (0.78)	42 (0.08)	484 (0.68)	46 (0.06)	27 (1.10)	1 (0.04)	9 (0.16)	0 (0.00)
P5	H3K27ac	465 (0.85)	80 (0.15)	552 (0.77)	92 (0.13)	40 (1.63)	3 (0.12)	10 (0.18)	0 (0.00)
P6	H3K27ac	2850 (5.20)	69 (0.13)	3436 (4.80)	77 (0.11)	168 (6.84)	2 (0.08)	62 (1.11)	0 (0.00)
P1	H3K4me3	366 (1.19)	41 (0.13)	507 (1.28)	53 (0.13)	21 (1.86)	8 (0.71)	27 (0.91)	2 (0.07)
P2	H3K4me3	660 (2.14)	185 (0.60)	946 (2.39)	226 (0.57)	38 (3.37)	12 (1.06)	61 (2.05)	14 (0.47)
P3	H3K4me3	872 (2.82)	75 (0.24)	1161 (2.93)	99 (0.25)	57 (5.05)	6 (0.53)	54 (1.82)	13 (0.44)
P4	H3K4me3	230 (0.74)	31 (0.10)	314 (0.79)	53 (0.13)	11 (0.98)	3 (0.27)	16 (0.54)	6 (0.20)
P5	H3K4me3	203 (0.66)	124 (0.40)	293 (0.74)	146 (0.37)	8 (0.71)	3 (0.27)	21 (0.71)	4 (0.13)
P6	H3K4me3	1489 (4.82)	69 (0.22)	2166 (5.47)	94 (0.24)	56 (4.96)	6 (0.53)	167 (5.62)	7 (0.24)

*Number of consensus peaks from chr1-22 that are present in at least two of the six T-PLL with a z-score >=3 (copy number gain) or <=-3 (copy number loss). The proportion over the total number of consensus peaks is shown in parentheses.

[+]For consensus peaks from chr1-22 that are present in at least two of the six T-PLL and three normal. These peaks were included in differential analysis between the 3 normal and 6 T-PLL using diffbind package.

§Number indicates differential peaks that overlap 100-kb windows with a z-score >=3 or <=-3. The proportion over the total number of differential peaks is shown in parentheses, separately for differential peaks with increased ("Up") and decreased ("Down") signal in T-PLL.
